# Interfacial Transition Zone in Rock–Backfill Composite Structures Under Thermal Action: Formation, Characterization, and Degradation

**DOI:** 10.3390/ma19194048

**Published:** 2026-09-22

**Authors:** Yuye Tan, Jiangwei Liu, Hongjiao Li, Ziyi Zeng

**Affiliations:** 1 State Key Laboratory of High-Efficient Mining and Safety of Metal Mines of Ministry of Education, University of Science and Technology Beijing, Beijing 100083, Chinam202520091@xs.ustb.edu.cn (H.L.); 17869412573@163.com (Z.Z.); 2 School of Resources and Safety Engineering, University of Science and Technology Beijing, Beijing 100083, China

**Keywords:** interfacial transition zone (ITZ), elevated curing, freeze–thaw cycles, micro-characterization, mechanical degradation

## Abstract

This review presents a systematic synthesis of the interfacial transition zone (ITZ) in rock–backfill composite structures under thermal action, focusing on (i) its formation mechanism, (ii) multi-scale characterization methodologies, (iii) thermally induced microstructural damage, and (iv) macroscopic interfacial mechanical degradation. A multi-tier literature retrieval protocol covering six databases (with foundational texts from 1945 and primary literature up to 2026) was employed to establish a rigorous core corpus. Furthermore, the micro-to-macro evolution laws under elevated curing temperatures, extreme high-temperature exposure, and freeze–thaw cycles are critically evaluated, and the limitations of current techniques and future research directions are discussed. This review provides a theoretical reference for ensuring interfacial stability in deep and special-environment backfill mining.

## 1. Introduction

Mineral resources form the material basis upon which human survival depends and are of great significance to the sustained and healthy development of the national economy [1]. Characterized by high safety, operating efficiency, and eco-friendliness, cemented backfill mining represents an essential trajectory toward green mining practices, thereby serving as the preferred extraction method for deep underground metal mines [2].

During backfilling mining, the surrounding rock and the backfill material form an interwoven composite structure. As a critical component—and indeed a permanent part—of the underground working, the mechanical properties and overall stability of this composite structure are crucial for controlling working pressure and maintaining the long-term stability of the underground working [3]. Furthermore, once the backfill material is delivered to the goaf, the relatively soft, paste-like cemented backfill is surrounded by hard rock with an irregular, uneven surface, forming a mesoscale interfacial transition zone (ITZ). This ITZ is essentially a heterogeneous cement-based composite material, and the formation and evolution of its complex, porous microstructure are highly dependent on the surrounding environment [4]. The physical properties, structural characteristics and mechanical properties of this composite material are influenced not only by its own composition (such as the cement-to-sand ratio and mass concentration) but are also closely related to the external ambient temperature; temperature has a decisive influence on its microstructural evolution and macroscopic mechanical properties [5].

As surface and shallow mineral resources become increasingly depleted, deep mining has emerged as the future trend in the mining industry. Currently, there are more than 110 mines worldwide operating at depths exceeding 1000 m, with some deep mines having reached depths of over 3000 m [6,7]. As mining depths increase, the temperature within the working face rises significantly due to the geothermal gradient, compounded by heat generated by machinery, personnel activities and blasting operations; the initial temperature in some deep mines abroad has already exceeded 80 °C [8,9]. Meanwhile, in high-altitude, cold regions of China such as Tibet, Qinghai and Xinjiang, as well as in permafrost mining areas in Northern Europe, Canada and Russia, mining workings are constantly exposed to complex operating conditions characterized by extremely low temperatures and even freeze–thaw cycles [10]. Temperature influences the micro- and nano-mechanical properties by regulating the hydration reactions, product composition and pore structure in the ITZ, thereby affecting the load-bearing capacity of the rock–backfill composite structure. Consequently, investigating the micro- to macro-scale mechanical evolution of the ITZ under the influence of temperature holds significant scientific and engineering value for assessing its long-term thermal stability.

To establish the academic necessity and novel positioning of this work, two recent reviews are most pertinent to the present study. Chen et al. [11] comprehensively reviewed ITZ identification, physical characteristics, and mechanical properties in conventional structural concrete, but their scope was confined to aggregate–paste interfaces under ambient environments, leaving the thermal degradation kinetics of mine backfill unaddressed. Wu et al. [12] synthesized the mechanical behavior and multi-physics modeling of surrounding rock–cemented paste backfill (CPB) composite structures yet treated the interface as a planar contact discontinuity without resolving the 15–50 μm ITZ or its temperature-dependent phase damage. The present review therefore fills the gap between these two syntheses: it is, to the authors’ knowledge, the first to systematically trace how thermal boundary conditions propagate from hydration chemistry, through pore-structure evolution, to macroscopic bond and shear degradation at the rock–backfill ITZ.

The remainder of this review is organized as follows: Section 2 outlines the systematic literature methodology and comparative framework. Section 3 details the formation mechanisms and initial characteristics of the ITZ. Section 4 evaluates multi-scale microstructural characterization techniques. Section 5 analyses the multi-scale mechanisms of microstructural evolution under thermal conditions. Section 6 addresses macroscopic mechanical degradation. Finally, Section 7 draws conclusions and proposes future research directions.

## 2. Systematic Literature Methodology, Bibliometrics, and Comparative Framework

### 2.1. Literature Search Protocol, Screening Logic, and Eligibility Criteria

To establish methodological transparency, reproducibility, and scholarly rigor, this review deploys a multi-tier literature retrieval protocol designed to capture foundational backgrounds, characterization methodologies, and thermal interfacial mechanisms without introducing retrieval bias. The detailed retrieval configuration is summarized in Table 1.

### 2.2. Bibliometric Landscape and Thematic Evolution

Following rigorous screening, deduplication, and full-text eligibility appraisal, a refined core corpus of 82 primary references was established. A bibliometric survey across this corpus reveals four well-defined thematic clusters (see Figure 1):

(1) Field Geothermal Background and Engineering Context (8.5%), defining the geothermal gradient, in situ stress fields, and ground-support imperatives across deep metal mines and frost-susceptible open-pit or permafrost environments;
(2) Microstructural Characterization Methodologies and ITZ Fundamentals (36.6%), documenting experimental benchmarks, resolution limits, and analytical models for BSE-SEM, nanoindentation, X-ray CT, and MIP, alongside foundational interface transition zone (ITZ) and pore-percolation theory across cementitious matrices;
(3) Thermally and Freeze–Thaw Induced Microstructural Phase Damage (23.2%), evaluating the sequential dehydration of ettringite (AFt), dehydroxylation of portlandite (CH), and structural collapse of calcium silicate hydrate (C-S-H) under extreme temperatures, alongside thermal expansion mismatch cracking and ice-phase crystallization fatigue under freeze–thaw cycles;
(4) Macroscopic Interfacial Mechanical Evolution and Comparative Synthesis (31.7%), quantifying normal tensile bond loss, shear stress degradation, cohesion softening, and friction angle shifts at rock–concrete and rock–backfill interfaces under extreme high-temperature or cyclic freeze–thaw conditions, and synthesizing findings against established macro-mechanical literature.

Geographically, experimental contributions on rock–backfill and rock–concrete interfaces are heavily centered in countries with extreme engineering settings, such as China, Canada, and Australia—regions directly confronting either extreme geothermal conditions in kilometer-deep mining or seasonal frost-heave hazards in open-pit and permafrost mining zones (see Figure 2). Historically, foundational studies prior to 2010 focused primarily on aggregate “wall effects,” hydration pore structures, and empirical shear testing at ambient temperatures. Between 2015 and 2026, the literature evolved substantially toward multi-scale coupled thermo-hydro-mechanical-chemical (THMC) simulations, grid-based nanoindentation phase mapping, micro-CT/NMR damage reconstruction, and the precise decoupling of early curing temperature acceleration from post-curing environmental exposure.

### 2.3. Comparison with Existing Reviews and Research Gaps

To establish the academic necessity and novel positioning of this work, the present study is benchmarked against the two most pertinent recent reviews in related domains: Chen et al. [11] and Wu et al. [12].

Chen et al. [11] conducted a comprehensive review on ITZ in cementitious materials, providing valuable insights into micro-imaging, nanoindentation mapping, and fracture mechanics. However, their scope was primarily confined to conventional structural concrete (aggregate–paste interfaces) under ambient environments, leaving the thermal degradation kinetics and unique rheological characteristics of mine backfill unaddressed. On the other hand, Wu et al. [12] synthesized the mechanical behavior and multi-physics modeling of surrounding rock–cemented paste backfill (CPB) composite structures from a macroscopic mining engineering perspective. Nonetheless, their focus remained predominantly at the stope and laboratory specimen scale, treating the interface as a planar contact discontinuity without resolving the 15–50 μm microstructural ITZ or its temperature-dependent phase-damage mechanisms.

To delineate the boundaries of existing syntheses and justify the present contribution, Table 2 compares these benchmark reviews across core analytical dimensions and identifies the specific knowledge gaps this paper addresses.

Through this comparative evaluation, three prominent research bottlenecks are identified:

(1) Scarcity of Direct Experimental Evidence for CPB–Rock Interfaces under Extreme Thermal Actions: While thermal damage mechanisms are extensively documented for standard structural concrete, direct experimental investigations isolating the 15–50 μm boundary layer between rock and cemented paste backfill (CPB) under extreme environments (e.g., deep geothermal heat or permafrost freeze–thaw cycles) remain severely limited. Consequently, current field assessments frequently rely on extrapolating chemical dehydration or frost-heave mechanisms from analogous shotcrete or concrete systems, leaving the actual phase-damage thresholds of the high-water-to-binder CPB interface largely unverified.
(2) Disconnect Between Microstructural Damage and Macroscopic Interfacial Mechanics: Microstructural observations (e.g., nanoindentation, BSE-SEM phase mapping) and macroscopic mechanical evaluations (e.g., direct shear, pull-out tests) are predominantly conducted as parallel, disconnected investigations. The academic community currently lacks a cohesive, cross-scale analytical framework that can quantitatively bridge temperature-induced micro-pore coarsening and mineral phase transitions directly to the macroscopic transition from cohesive bond failure to frictional slip.
(3) Oversimplification of In Situ Environmental and Morphological Boundary Conditions: Current ITZ characterizations are largely performed on idealized laboratory specimens featuring simplified thermal paths and artificially flattened rock surfaces. This reductionist approach fails to capture the multi-physics reality of actual underground stopes, where the interfacial zone evolves under the synergistic, coupled influence of blast-induced irregular surface roughness, high in situ consolidation pressures, and dynamic geothermal gradients.

### 2.4. Cross-Scale Review Framework

To resolve the highlighted bottlenecks and bridge the gap between microscopic alterations and macroscopic structural safety, this review adopts a cross-scale causal synthesis framework. This framework systematically maps thermal boundary conditions directly to chemical phase changes, pore-structure coarsening, and macro-mechanical failure through three distinct pathways:

(1) Micro-Scale Chemical and Mineral Phase Transformation: Elevated curing accelerates cement hydration kinetics, temporarily boosting early C-S-H precipitation. Conversely, sustained high-temperature thermal exposure initiates sequential dehydration (AFt decomposition at 60–80 °C, interlayer water release from C-S-H at 180–300 °C, and CH dehydroxylation above 450 °C). Under freeze–thaw cycles, pore water ice-phase transitions induce localized crystallization pressure and cryogenic suction.
(2) Meso-Scale ITZ Pore Topology and Elastic Property Evolution: Thermal decomposition and differential thermal expansion coefficients between rock and cement paste generate localized boundary shear stresses and microcracking. Under frost action, repetitive ice lens growth broadens the ITZ geometric thickness and degrades nanoindentation hardness.
(3) Macro-Scale Bond and Shear Degradation: Microstructural pore networks and microcracks govern the macroscopic mechanical capacity, controlling normal tensile bond failure, cohesion loss, internal friction angle variations, and the transition from adhesive failure to brittle interfacial slip.

## 3. The Mechanism of ITZ Formation and Its Initial Characteristics

### 3.1. Genesis Mechanisms: Particle Packing ‘Wall Effect’ and Bleeding Channels

ITZ is a specific zone within cement-based composites located between the aggregate (or rock) and the cement paste, typically with a thickness of 15–50 μm. Microstructurally, the ITZ exhibits significant heterogeneity, with a porosity higher than that of the matrix; calcium hydroxide (CH) crystals are enriched and arranged perpendicular to the aggregate surface, whilst the calcium silicate hydrate (C-S-H) gel has a lower density, forming a double-layer structure [13]. The mechanical properties of this zone are generally weaker than those of the matrix; it acts as a weak link where internal stresses concentrate and damage initiates, exerting a decisive influence on the macroscopic strength and durability of the composite material [14]. In rock–backfill composite structures (see Figure 3), the ITZ serves as the key link connecting the surrounding rock and the backfill (see Figure 4). A systematic elucidation of the formation mechanism and initial characteristics of the ITZ is an essential foundation for understanding the evolution of the interface under the influence of temperature.

The formation of ITZ is a complex process driven jointly by physical constraints, chemical reactions and multiphase fluid migration. Its origin was initially attributed to a geometric interference phenomenon, namely the ‘wall effect’, a classic concept in academic circles. This theory was first systematically proposed by Powers [16] in 1958, and its core mechanism can be vividly summarized as the ‘queuing theory’ of particle packing: when randomly distributed cement particles approach a hard, flat rock surface, their physical diameter limits prevent them from achieving the dense interlocking arrangement characteristic of the matrix (see Figure 5).

Maso [17] points out that the rock surface alters the gradient of the random particle distribution, causing a sharp drop in the density of large cement particles within a 20–50 μm range at the interface; the excess space is filled with free water, resulting in the formation of a thin, low-density paste layer with a high water-to-cement ratio even before hydration begins. This phenomenon is the physical cause of the ITZ’s poor mechanical properties. Upon entering the hydration reaction stage, this initial physical disparity in arrangement evolves into an uneven distribution of chemical products.

During the hydration stage, initial differences in physical arrangement evolve into an uneven distribution of chemical products. Scrivener et al. [13] found, through backscattered electron imaging, that regions within the ITZ with a high water-to-cement ratio provide ample space for the development of calcium hydroxide (CH) and ettringite (AFt) crystals, with CH crystals tending to grow in an orientation anchored to the rock surface. Bentz [18] confirmed via a CCBM model that layered CH crystals are oriented parallel to the rock surface; this preferential orientation reduces the effective bonding area at the interface. Combined with low shear resistance, this renders the ITZ the primary failure path under stress. Ollivier [19] further noted that this oriented growth is regulated by the chemical potential of the rock’s mineral components, exhibiting micro-scale anisotropy.

From the perspective of pore evolution, the spatial distribution of the final ITZ structure is non-uniform, with multi-scale pore networks interweaving and interconnecting. Winslow’s [20] ‘seepage theory’ posits that large pores within the ITZ interconnect once they reach a critical volume, forming continuous multi-scale pore defect channels. In a mine backfill environment, this process is strongly influenced by the bleeding of the grout. Research by Cui et al. [21] indicates that fluid migration triggered by the release of excess pore water pressure during the consolidation of the backfill grout tends to result in ‘micro-convergence’ along the ITZ, eroding water channels or micro-pores.

In summary, the ITZ is a transitional interface zone approximately 15–50 μm thick between the rock and the backfill material; it exhibits mechanical properties weaker than those of the matrix and displays significant heterogeneity. This heterogeneity constitutes a weak link in mechanical load-bearing capacity and is a key site for damage accumulation in mine backfill environments. Elucidating the mechanisms governing the evolution of ITZ characteristics is of great significance for the strength design of backfill materials and the control of their long-term stability.

### 3.2. Coupled Effects and Implications for Interfacial Mechanical Baselines

The microstructural genesis and initial bonding performance of the ITZ in rock–backfill composite structures are highly sensitive to downhole environmental conditions during the early curing stage. Among all environmental factors, curing temperature, ambient moisture, and in situ stress states play dominant roles in dictating the early hydration kinetics and the resulting heterogeneity of the interface [22].

(1) Thermal Boundary (Curing Temperature): Temperature acts as a dual-edged regulator during early-stage curing. Moderate temperature elevations (20–60 °C) accelerate the dissolution of clinker phases and promote early nucleation of hydration products such as calcium silicate hydrate (C-S-H) and ettringite (AFt) [23,24]. However, excessively high early curing temperatures induce a rapid, heterogeneous precipitation around unhydrated cement grains, forming a dense inner shell that hinders subsequent ion diffusion. This leads to a coarser pore network and higher long-term porosity within the 15–50 μm ITZ domain [25,26].
(2) Moisture Availability and Relative Humidity (RH): Downhole moisture gradients strongly influence the micro-bleeding and self-desiccation behaviors at the rough rock-grout boundary [27]. High ambient humidity sustains continuous hydration across the interface, whereas low RH conditions generate severe capillary suction and micro-shrinkage, triggering early-age microcracking specifically localized within the weak ITZ [28].
(3) Coupled Effects and Implications for Interfacial Mechanical Baselines: These environmental factors during curing directly govern the baseline mechanical properties evaluated later in macroscopic tests. For instance, moderate thermal curing (35 °C) optimizes short-term chemical bonding and increases 7-day interfacial shear strength. However, the concurrent microstructural coarsening induced by thermal acceleration compromises long-term durability, explaining the strength crossover observed in Section 6.1, where 35 °C-cured interfaces outperform at 7 days but underperform at 28 days compared to standard ambient curing (20 °C) [29]. Incorporating these curing-stage environmental factors bridges the gap between microscopic formation mechanisms and the macroscopic mechanical degradation trends observed in deep mining environments.

## 4. ITZ Microstructural Characterization Technology

As the weakest link in cement-based composite materials, the microstructure and mechanical behavior of the interface zone (ITZ) directly determine the macroscopic properties of the material. However, due to the small spatial scale and high structural heterogeneity of the ITZ, its precise detection and characterization have long been major challenges in this field of research. This section reviews the four most widely used ITZ characterization techniques, summarizing and analyzing the advantages and limitations of each method.

### 4.1. Scanning Electron Microscope–Backscattered Electron Imaging (BSE-SEM)

BSE-SEM is the most established technique for characterizing the microstructure of the ITZ; its physical basis lies in the principle of atomic number contrast imaging: elements with high atomic numbers appear as bright gray tones, whilst those with low atomic numbers appear as dark black. In cementitious materials, this characteristic results in clear gray-scale gradients for calcium hydroxide (CH) crystals, calcium silicate hydrate (C-S-H) gel, unhydrated clinker particles and pores (see Figure 6), thereby enabling the identification and quantitative analysis of mineral phases.

In 1984, Scrivener and Pratt [30] were the first to observe, using optical microscopy combined with fluorescent resin impregnation techniques, that the porosity of the ITZ was significantly higher than that of the cementitious matrix; however, resolution limitations prevented them from classifying the hydration products in detail. With improvements in SEM resolution and advances in image processing algorithms, BSE-SEM gradually established itself as the mainstream technique. In 2004, Scrivener [31] systematically outlined the methodology of backscattered electron imaging for the quantitative characterization of cementitious materials, establishing a comprehensive analytical framework ranging from image acquisition and gray-scale threshold segmentation to the statistical analysis of phase volume fractions. Diamond et al. [32] demonstrated, using high-resolution BSE images, that the traditional assumption of a homogeneous, thin layer was overly idealized. Furthermore, Diamond [33] specifically discussed considerations regarding the application of image analysis techniques in ITZ research, pointing out that the quality of epoxy resin impregnation and polishing during sample preparation has a significant impact on quantitative results. In 2004, Stutzman [34] provided a further detailed introduction to the application of SEM in the microstructural imaging of cement-based materials, emphasizing the critical influence of sample preparation parameters on imaging quality.

For ITZ analysis, however, BSE-SEM is strictly limited to two-dimensional cross-sectional observations; it cannot reconstruct three-dimensional pore network connectivity directly and remains highly sensitive to preparation artifacts such as polishing relief and resin plucking [24,25].

### 4.2. Nanoindentation Technology

For a long time, research into the mechanical properties of the ITZ has been constrained by its extremely small spatial scale; conventional macroscopic tests have been unable to isolate the ITZ for independent study, and the spatial resolution of traditional hardness testing has been insufficient to localize the ITZ region. Nanoindentation technology has resolved the challenge of quantitatively characterizing the mechanical properties of ITZs. By recording the load–displacement curve of a diamond indenter during the loading–unloading process and calculating the material’s elastic modulus and hardness values based on the model proposed by Oliver and Pharr [35] in 1992 (see Figure 7), this technique extends the spatial scale of mechanical testing to the sub-micrometer level, thereby enabling the direct mechanical characterization of ITZs.

However, there is considerable mechanical heterogeneity within the ITZ, and single or random indentation points are often subject to significant positioning errors; random indentations may land on unhydrated particles or low-density C-S-H or CH crystals, making the results difficult to interpret. To overcome this challenge, Constantinides and Ulm [36] proposed a grid-based nanoindentation technique in 2007. By establishing a dense array of indentations in the target region and combining this with statistical clustering algorithms, they inverted vast amounts of mechanical data into the characteristic properties and volume fractions of different hydration products, thereby establishing, for the first time, a precise quantitative correlation between ITZ composition and performance. In earlier work in 2006, Constantinides et al. [37] had already laid the statistical foundations for grid-based indentation and proposed analytical methods suitable for multiphase composite materials. Mondal et al. [38] further developed reliable nanoindentation testing techniques, enhancing the repeatability and reliability of test results by optimizing loading protocols and data processing workflows. In 2008, Miller et al. [39] specifically investigated the effects of surface roughness in nanoindentation of cement paste, pointing out that the quality of sample preparation has a decisive influence on the test results.

For ITZ analysis, however, nanoindentation suffers from finite indenter footprint and size effects [39], and severe boundary/edge effects occur when the indenter approaches the rough aggregate surface, leading to local measurement distortions.

### 4.3. X-Ray Computed Tomography (X-Ray CT)

Whilst BSE-SEM and nanoindentation each have their own strengths in terms of compositional and mechanical characterization, both are limited to two-dimensional observation. Given that the permeability of the ITZ is highly dependent on the three-dimensional connectivity of the pores, X-ray computed tomography (X-ray CT) technology bridges the gap between two-dimensional and three-dimensional imaging. X-ray CT utilizes the differences in attenuation of X-rays of different energies by the sample to perform tomographic imaging; by rotating the sample, multi-angle projection data are acquired, which are then reconstructed using algorithms to obtain three-dimensional volume data. As early as 1991, Bentz and Garboczi [40] established a three-dimensional digital-image-based model of cement hydration and percolation; although not a CT study, this work pioneered the quantitative analysis of 3D phase connectivity that later X-ray CT experiments made directly observable (Figure 8).

In 2003, Landis et al. [41] applied X-ray CT to the microstructural analysis of concrete, demonstrating the unique advantages of CT technology in capturing three-dimensional crack networks. Bentz et al. [42] further applied X-ray CT to the study of the microstructure and transport properties of porous building materials, establishing a complete analytical workflow from image acquisition to the prediction of transport properties. Batista [43] provided a systematic review of the progress in the application of X-ray micro-computed tomography in the study of cementitious materials, noting that the technology is expanding from static structural characterization to the monitoring of dynamic processes. Dong et al. [44] applied CT technology to the study of self-healing concrete systems, demonstrating its applicability to complex engineering problems. Kim et al. [45] utilized X-ray CT to evaluate the evolution of the pore structure and cracking behavior of cement paste following exposure to high temperatures, thereby expanding the application of CT in research into extreme environments.

For ITZ analysis, however, high equipment costs, lengthy scanning cycles, and insufficient spatial resolution for nanoscale gel pores or fine interlayer changes within C-S-H restrict its standalone application for thin ITZ domains.

### 4.4. Mercury Intrusion Porosimetry (MIP)

In quantitative studies of ITZ pore structure, the mercury intrusion porosimetry (MIP) is currently one of the most commonly used techniques. Based on the Washburn equation, MIP utilizes the non-wetting behavior of mercury on solid surfaces; by gradually increasing the external pressure, mercury is forced into the pores of the sample, and the pore size distribution is calculated from the relationship between pressure and the volume of mercury intruded (see Figure 9).

As early as 1970, Winslow and Diamond [46] applied the MIP system to the analysis of the porosity of cement paste. They systematically investigated the evolution of the pore structure during the hydration process and proposed the concept of ‘threshold pore size’, defined as the minimum connected pore size at which mercury rapidly penetrates the sample. Since then, MIP has been widely used in comparative studies of the pore structures of the ITZ and the matrix, providing a wealth of experimental data to elucidate the pore-related mechanisms underlying the high permeability of the ITZ.

Although MIP offers advantages such as ease of operation and good reproducibility, its limitations have also been the subject of widespread debate. Diamond [47], based on the application of MIP in cementitious materials, pointed out that the high-pressure process may lead to microstructural damage. Igarashi and Nishikawa [48] compared the results of MIP and BSE-SEM image analysis in characterizing the pore structure of cement paste and found that the two methods showed good consistency in the identification of coarse capillaries, but there were significant differences at the gel pore scale. Research by Muller and Scrivener [49] indicates that capillaries form a continuous network within the sample and are connected to the exterior; consequently, the fact that MIP measures capillaries before penetrating gel pores mitigates the ‘inkwell’ effect to some extent. However, this conclusion is primarily based on cement paste with a water-to-binder ratio of 0.40, and its applicability to systems with low water-to-binder ratios or those containing mineral admixtures remains to be verified.

For ITZ analysis, however, MIP cannot spatially isolate the thin ITZ from the bulk matrix; it therefore characterizes the consequence of overall composite damage and bulk porosity rather than isolating the ITZ itself, and high-pressure intrusion can induce artificial microstructural damage in weak ITZ regions [47].

### 4.5. Critical Synthesis and Methodological Comparison of Characterization Techniques

Due to the narrow spatial scale (15–50 μm) and high structural heterogeneity of the ITZ in rock–backfill composite systems, relying on a single analytical tool is insufficient to comprehensively capture its multi-scale evolution. A critical synthesis of the four primary characterization techniques—BSE-SEM, nanoindentation, X-ray CT, and MIP—reveals distinct trade-offs regarding their spatial resolution, measurable parameters, and intrinsic limitations, as systematically summarized in Table 3.

**Table 3:** Critical comparison of micro-characterization techniques for the ITZ in rock–backfill composite structures.

Technique	Spatial Resolution	Measurable Parameters	Key Advantage	Intrinsic Limitations for ITZ Analysis
BSE-SEM	Sub-micron	Phase volume fractions, local porosity gradients, microstructural morphology, elemental distribution (via EDS)	High-resolution 2D visualization and phase identification	Two-dimensional cross-sectional restriction; highly sensitive to polishing and epoxy impregnation artifacts
Nanoindentation	Nanometer scale (indenter footprint ~1–3 µm, spacing ~1 µm)	Local elastic modulus (E), hardness (H), phase-specific mechanical properties via grid deconvolution	Direct quantitative mechanical characterization at sub-micrometer scale	Size effects, severe boundary and edge effects near rough aggregate surfaces; sensitive to surface roughness
X-ray CT	Sub-micron to micrometer (0.5–5 µm)	3D pore network topology, microcrack propagation, spatial connectivity, volumetric evolution	Non-destructive 3D visualization of internal microstructures and crack networks	High equipment cost, lengthy scanning cycles, and insufficient resolution to capture nanoscale gel pores
MIP	Meso-to-macro pore scale (nanometer to micrometer range, typically 3.5 nm to 200 μm)	Total porosity, threshold pore size, critical pore diameter, cumulative pore volume distribution	Easy operation and good reproducibility for global pore network connectivity	Assumes cylindrical pore models; high-pressure intrusion causes structural damage; unable to spatially isolate the thin ITZ

As synthesized above, BSE-SEM and nanoindentation provide exceptional high-resolution compositional and mechanical insights in 2D space, but they lack volumetric context. Conversely, X-ray CT and MIP reveal 3D pore architecture and connectivity at the expense of nanoscale phase resolution. Therefore, to overcome individual methodological bottlenecks, future research on rock–backfill ITZs must implement a multi-scale collaborative framework combining non-destructive 3D imaging with high-resolution local mechanical and chemical mapping.

## 5. Multi-Scale Mechanisms of Microstructural Evolution in the ITZ Under Thermal Conditions

The ITZ is the primary locus for environmentally induced damage in rock–backfill composite structures [50]. To overcome the limitations of descriptive summaries, this section establishes a micro–meso–macro causal framework linking initial hydration chemistry to pore evolution, and subsequently to ITZ stiffness degradation.

### 5.1. Microstructural Evolution During Elevated-Temperature Curing

Elevated curing temperatures act simultaneously on unhydrated cement particles and early hydration kinetics. Moderate thermal curing (e.g., 20 °C to 60 °C) accelerates the dissolution of clinker phases and promotes early chemical cementation. Direct experimental investigations on the CPB–rock interface by Zhang et al. [51] demonstrated that chemical bonding and mechanical interlocking dominate microstructural development at these early stages (see Figure 10), establishing a highly interconnected initial matrix before the onset of external mechanical loads.

However, this kinetic acceleration presents a critical microstructural trade-off. As revealed by Fang and Fall [52] through thermogravimetric and mercury intrusion porosimetry (MIP) analyses, the accelerated kinetics are accompanied by a notable coarsening of the pore structure. This observation aligns with the well-established “inner-product shell” consensus in cement chemistry, where rapid precipitation around unhydrated grains hinders subsequent ion diffusion, ultimately retarding the continuous hydration process. The consequence is a coarser pore network and higher long-term porosity specifically localized within the 15–50 µm ITZ domain. Providing theoretical substantiation, the fully coupled THMC model established by Cui and Fall [21] mathematically projects that the internal heat of hydration released during backfill hardening creates complex temperature fluctuations, further exacerbating this early-age microstructural heterogeneity.

### 5.2. Microstructural Damage Mechanisms Under High-Temperature Exposure

When an already-cured rock-binder composite is exposed to high temperatures, thermal damage manifests through two primary, coupled mechanisms: the staged thermal decomposition of hydration products and physical microcracking induced by thermal expansion mismatches.

#### 5.2.1. Dynamic Thermal Decomposition of Hydration Phases

The degradation of the ITZ under high-temperature exposure is governed by the sequential dehydration of its mineral phases. These decomposition stages are highly dynamic and depend significantly on heating rates, moisture conditions, and sealing. Under initial heating (30 °C to 100 °C), free water evaporation generates capillary negative pressures that nucleate primary microcracks within the highly porous ITZ. Between 60 °C and 80 °C, ettringite (AFt) undergoes initial dehydration. Wang et al. [53] identified 70 °C to 75 °C as the critical window for AFt to monosulfoaluminate (AFm) transformation (see Figure 11). While Afflerbach et al. [54] noted this crystal strain can be partially reversible under immediate rehydration, sustained thermal exposure permanently loosens the initial ITZ architecture.

As temperatures elevate further (180 °C to 300 °C), the dominant binding phase, C-S-H gel, experiences severe interlayer dehydration. Ma et al. [55] and Lu et al. [56] reported significant interlayer contraction near 200 °C and collapse of the layered structure by approximately 400 °C under their specific heating regimes; molecular dynamics simulations indicate that the high Ca/Si ratio typical of ITZ C-S-H renders it intrinsically less thermally stable than bulk matrix C-S-H [57]. At extreme temperatures beyond 450 °C, calcium hydroxide (CH) undergoes dehydroxylation, and under continued exposure toward 700 °C, C-S-H decomposes completely.

#### 5.2.2. Thermal Expansion Mismatch

Concurrently with chemical dehydration, severe geometric thermal mismatches drive physical ITZ degradation. Natural rocks typically possess a thermal expansion coefficient of 5 × 10^−6^/°C to 12 × 10^−6^/°C, whereas highly porous cementitious matrices exhibit much higher expansion rates. This fundamental material difference generates intense local boundary shear stresses during heating and subsequent cooling cycles. When these localized thermal stresses exceed the inherently low fracture toughness of the ITZ, stress concentrations arise at the tips of the chemically weakened microcracks. This synergistic coupling of chemical softening and physical expansion forces isolated defects to coalesce, which can be expected to initiate macroscopic interfacial debonding [58].

### 5.3. Mechanisms of ITZ Deterioration Under Freeze–Thaw Cycling

Unlike the chemically driven degradation of high-temperature exposure, cyclic freeze–thaw actions degrade the ITZ through repetitive physical fatigue induced by ice-phase transitions and cryogenic frost heave. Early hypotheses by Powers [59,60] attributed this damage to hydrostatic and osmotic pressures within standard concrete. However, modern micro-characterization reveals that the high initial porosity of the ITZ acts as the primary preferential pathway for moisture accumulation and supercooled water migration (see Figure 12) [61].

When localized crystallization expansion forces and cryogenic osmotic suction exceed the matrix’s tensile strength, repetitive micro-tearing occurs. Nanoindentation tests by Pan et al. on sandstone–concrete interfaces demonstrated significant decreases in local elastic modulus and hardness [62]. Reported ITZ widening under freeze–thaw varies considerably with material and cycle regime: from 20 µm to 65 µm in ultra-high-performance concrete (UHPC) [63], and from 25 µm to 50 µm with a 50% hardness loss after 1500 cycles in conventional concrete [64]. Whether a residual-strength plateau follows the rapid initial degradation is likewise system-dependent, and consistent cycle-range thresholds remain to be established.

Furthermore, this pore-scale damage is exacerbated by macroscopic frost-heave mismatches. Differences in volumetric expansion and contraction between rigid rock aggregates and the highly saturated paste generate severe peeling stresses. Song et al. confirmed that this differential expansion continuously drives delamination precisely along the weakened ITZ, creating a highly sensitive zone with elevated porosity immediately adjacent to the rock surface [65].

In summary, the microstructural evolution of the ITZ is strictly governed by mechanisms tailored to the applied thermal environment. Elevated-temperature curing accelerates early hydration kinetics but coarsens long-term pore structures. High-temperature exposure drives a coupled degradation pathway defined by phase dehydration (AFt, C-S-H, CH) and thermal expansion mismatch. Conversely, freeze–thaw cycling inflicts cumulative physical fatigue via localized ice crystallization and differential frost heave, where void accumulation progressively tears and widens the ITZ boundary.

Of the mechanisms reviewed above, only the curing-temperature trade-off [51,52] and early bonding evolution are directly evidenced on CPB–rock interfaces; phase decomposition thresholds, expansion mismatch cracking, and freeze–thaw void accumulation are extrapolated from concrete, mortar, and shotcrete systems and remain to be verified on dedicated CPB–rock specimens.

## 6. Macroscopic Mechanical Degradation of ITZ-Driven Interfaces

The microstructural pore networks and chemical phase transformations directly govern macroscopic mechanical capacity, controlling normal tensile bond failure, cohesion loss, and the transition to brittle interfacial slip. The macroscopic responses observed in existing studies vary significantly depending on the thermal protocols applied. To construct a rigorous evaluation, the following analysis categorizes macroscopic mechanical degradation according to specific thermal regimes. To eliminate ambiguous extrapolations, the supporting literature is critically appraised by distinguishing direct rock–CPB experimental evidence from indirect inferences derived from analogous rock–concrete or shotcrete interfaces, alongside theoretical hypotheses based on fracture modeling.

### 6.1. Macroscopic Evolution Under Elevated Curing Temperatures

Elevated curing temperatures fundamentally alter early bond development and shear strength. Direct experimental evaluations of the CPB–rock interface by Fang and Fall [52] revealed a critical temporal trade-off: samples cured at 35 °C exhibited maximum cohesion and internal friction at 7 days due to accelerated chemical cementation (see Figure 13). However, by 28 days, this strength was eclipsed by samples cured at 20 °C. The initial thermal acceleration triggers subsequent pore coarsening, ultimately compromising the long-term interfacial shear strength [52]. These curing temperature effects represent a kinetic structural trade-off entirely distinct from the destructive degradation seen in extreme high-temperature exposure.

### 6.2. Macroscopic Degradation Under High-Temperature Exposure

When an already-cured interface is subjected to severe heat, normal bond and shear strengths deteriorate systematically, driven by the chemical and physical degradation established in Section 5.2. Because the mechanical response is strictly dictated by the testing environment, it is imperative to isolate in situ hot-state testing from residual testing performed after cooling regimens.

#### 6.2.1. In Situ Hot-State Testing

Evaluating the interface directly at elevated temperatures highlights the immediate impact of geothermal environments prior to any cooling-induced thermal shock (see Table 4 for a summary of high-temperature evidence categorized by detailed thermal protocols). Investigating analogous shotcrete–rock interfaces, Tong et al. [66] found that in situ shear strength is highly moisture-dependent when tested directly between 20 °C and 100 °C. Peak shear strength occurred at 60 °C under 55% relative humidity, but shifted to 80 °C under 95% humidity. Similarly, Hu et al. [67] noted in hot-state evaluations that higher initial exposure temperatures amplify post-peak softening characteristics in shear stress-displacement responses, highlighting how heat immediately alters the ductility of the interface.

#### 6.2.2. Residual Testing After Cooling

Most extreme high-temperature studies utilize residual testing, which inherently incorporates unquantified thermal contraction damage during the cooling phase. Consequently, contrasting with the in situ hot-state evaluations discussed in Section 6.2.1, residual test results must be interpreted with caution when assessing true downhole thermal-stress responses. Studies on analogous systems, such as recycled concrete with steel sections by Chen et al. [68], show that residual ultimate bond strength drops precipitously by 32.8% to 79.7% as exposure temperatures rise from 200 °C to 600 °C. Similarly, Zhang et al. [69] demonstrated via pre-heating treatments that peak shear strength, cohesion, and friction angles decline proportionally with temperature elevation.

Extreme temperatures induce a qualitative shift in the mechanical failure mode. In residual direct shear studies, Chen et al. [70] observed that the failure plane progressively migrates toward, and eventually localizes at, the rock–concrete interface as temperatures range from 20 °C to 450 °C (see Figure 14). Cyclic shear studies by Zhang et al. [71] and variable-temperature tests by Hu et al. [72] further revealed that severe dehydration of the ITZ forces the failure mechanism to transition from mixed internal and interfacial failure to sudden, brittle planar slip (see Figure 15). The applicability of these structural concrete findings to actual CPB–rock systems—which possess much higher water-to-cement ratios and lack the interlocking mechanisms of coarse aggregates—remains a theoretical hypothesis requiring specific future empirical validation.

**Table 4:** High-temperature evidence categorized by detailed thermal protocol.

Source	Interface	Curing Age	Target Temperature/Exposure	Heating Rate & Dwell	Moisture Condition	Cooling & Testing	Observed Response
Fang & Fall [52]	CPB–Rock	7 & 28 Days	2, 20, 35 °C	-	Sealed	Tested at curing temp (in situ)	35 °C maximized 7 d cohesion, but accelerated pore coarsening reduced 28-day shear strength.
Tong et al. [66]	Shotcrete–Rock	>28 Days	20–100 °C	Controlled environment	55% & 95% RH	Tested directly at designated temp (Hot-State)	Peak shear strength is moisture-dependent, occurring at 60 °C (55% RH) and 80 °C (95% RH).
Chen et al. [68]	Steel–recycled concrete	>28 Days	200–600 °C	Furnace heating	Ambient	Natural cooling; residual testing	Residual ultimate bond strength drops precipitously by 32.8% to 79.7%.
Chen et al. [70]	Rock–Concrete	>28 Days	20–450 °C	5 °C/min; 2 h dwell	Ambient	Natural cooling; residual shear testing	Failure plane progressively migrates to the rock–concrete ITZ above 300 °C.

### 6.3. Macroscopic Deterioration Under Freeze–Thaw Cycling

Unlike the chemically driven failure of thermal exposure, freeze–thaw cycling inflicts a progressive, cumulative linear decline on interfacial mechanics. Direct shear tests by Wang et al. [73] and bond strength degradation models calibrated by Shen et al. [74] bond strength model on rock-mortar interfaces confirm that interfacial shear strength decreases linearly with cycle counts. However, interfacial geometric roughness provides vital structural protection, acting to preserve residual friction angles even as chemical cohesion is severely eroded.

A critical evaluation of existing literature, however, reveals that macroscopic degradation trajectories vary substantially depending on the specific interface systems, material compositions, and testing protocols employed, precluding the application of a single universal pattern. While certain studies report an approximately linear reduction in shear strength over specific cycle ranges, others highlight a distinct non-linear evolution—typically characterized by a rapid initial deterioration during early cycles driven by capillary freezing and microcracking, followed by a transition toward a slower rate of decline or a residual stabilization phase.

To further qualify these discrepancies, this divergence between a continuous linear decline and a two-stage response stems from several interrelated micro-environmental and structural factors. First, the initial porosity and spatial heterogeneity of the ITZ, dictated by local water-to-binder ratios and bleeding channels, determine the volume and connectivity of space available for water migration and supercooled water accumulation. In systems with higher initial connectivity, cyclic ice-crystal growth and cryogenic suction rapidly tear the weak ITZ, triggering the steep initial drop in bond strength within the early freeze–thaw cycles. Second, the mechanical response is strongly regulated by morphological characteristics and stiffness mismatches. Systems incorporating robust mechanical interlocking (such as rough, natural fracture surfaces) or crack-bridging reinforcements (e.g., PVA fibers) exhibit a more pronounced residual stabilization phase. Once the initial weak ITZ accommodates the primary volumetric expansion, frictional resistance governed by surface roughness and clamping normal stress takes over, preventing catastrophic continuous collapse.

Kai et al. [75] tracked porosity increases during early cycles, while Liang et al. [76] found in analogous FRP-concrete systems that fracture energy declined sharply within the first 100 cycles, after which the rate of decline decelerated markedly, approaching a quasi-plateau (see Figure 16). Similar progressive declines in compressive and bond strengths were quantified in composite columns by Wang et al. [77] and Li et al. [78].

For rock-cement interfaces, complementary studies by Shen et al. [79,80] utilized NMR tracking to reveal that early-stage capillary freezing leads to rapid cohesion loss. As shown by Pan et al. [62], continuous capillary freezing and osmotic tearing steadily deplete the volume fractions of load-bearing C-S-H phases over time. To mitigate this continuous peeling, Wang et al. [81] demonstrated that PVA fibers can bridge localized microcracks to enhance shear durability, while Zhou et al. [82] established a fracture mechanics zone model predicting service life, providing a robust theoretical framework for evaluating long-term interfacial stability in cold regions.

To systematically synthesize the evidence quality across these diverse testing conditions, Table 5 categorizes the key literature, explicitly identifying the material systems and qualifying their evidence levels and extrapolability constraints.

In summary, the macroscopic mechanical degradation of the ITZ is inextricably linked to its microstructural evolution, strictly governed by the applied thermal regime. Elevated curing temperatures present a temporal trade-off, enhancing early-age chemical bonding while ultimately compromising long-term shear strength. High-temperature exposure of cured systems results in systematic cohesion loss and a critical transition to brittle interfacial slip above 300 °C, driven by severe phase dehydration and thermal expansion mismatch. Meanwhile, cyclic freeze–thaw actions induce a cumulative, fatigue-driven linear decline in normal bond and shear parameters, characterized by rapid initial deterioration followed by potential residual stabilization. By synthesizing direct observations, extrapolating mechanistic similarities from analogous materials, and applying theoretical models, a comprehensive understanding of rock–backfill thermal stability is established, outlining clear pathways for future empirical validation in deep mining environments.

## 7. Conclusions and Perspectives

This review provides a comprehensive overview of the current state of knowledge on the formation mechanisms, multi-scale characterization methodologies, thermally induced microstructural damage, and macroscopic mechanical degradation of the ITZ in rock–backfill composite structures. The factors influencing the physical, microstructural, and mechanical properties of the ITZ are evaluated, encompassing curing temperatures, elevated thermal exposure regimes, downhole moisture gradients, and freeze–thaw cycles. These factors can be classified into chemical, physical, and environmental drivers according to their influence on the initial heterogeneity and subsequent degradation of the ITZ, as detailed throughout this work. Based on a rigorous synthesis of extensive experimental, theoretical, and numerical literature, the following conclusions can be drawn:

(1) Accurate characterization of the physical and microstructural features of the ITZ is the prerequisite for implementing reliable numerical simulations and assessing long-term stability in deep mining, especially regarding its 15–50 µm thickness gradient and local elastic modulus. These properties provide valuable insights for refining ITZ modeling in rock–backfill composite structures for subsequent investigations. However, in most existing studies, these features are simply determined as constant parameters or neglected by treating the interface as a planar discontinuity, which makes it difficult to fully reflect the influence of thermal boundary variations and microstructural heterogeneity on the macro-mechanical behavior. Therefore, introducing more advanced multi-scale numerical models that incorporate the gradient distribution of physical features and thermal phase damage to simulate the damage accumulation process accurately may be a meaningful direction in the future. Specifically, ITZ thickness and porosity evolution under thermal action should be quantitatively linked to localized mineralogy and bleeding channels, following statistical distribution patterns.
(2) Although analytical models considering the existence of the ITZ have been extensively studied in general cementitious systems, most have focused on estimating bulk properties without resolving the temperature-dependent phase transformations and stress redistribution within the thin ITZ region. As highlighted by the current literature limitations, the uneven distribution of thermal stresses and strain gradients within the ITZ, driven by thermal expansion mismatches between hard rock and soft paste, is still not well explained. Therefore, the development of an analytical model that considers non-uniform thermal stress fields, sequential dehydration kinetics (such as AFt decomposition at 60–80 °C and C-S-H breakdown), and varying bonding strength remains an important topic for future research.
(3) The microstructure and physical properties of the ITZ are significantly influenced by multiple environmental and compositional factors. Among these, early curing temperature, sustained thermal exposure, and cyclic freeze–thaw actions directly affect the morphological features and phase composition of the ITZ by accelerating initial hydration kinetics or inducing severe microstructural damage. Elevated curing (e.g., 35 °C) enhances early chemical cementation and 7-day cohesion but triggers subsequent pore coarsening that eclipses long-term strength. Extreme high temperatures induce a critical transition to brittle planar slip above 300 °C due to phase dehydration, while freeze–thaw cycles drive cumulative physical fatigue and cryogenic void accumulation, leading to linear declines in interfacial shear strength. Optimizing the initial mix design, incorporating reinforcing fibers (such as PVA fibers), and controlling thermal boundary conditions are crucial for mitigating these degradation processes.
(4) Improving the ITZ microstructure and mechanical stability is a feasible strategy for enhancing the long-term load-bearing capacity and safety of rock–backfill composite structures in deep mining and cold environments. Improvements can be achieved through the precise control of curing protocols, the addition of mineral admixtures, and the implementation of geometric interlocking. These approaches aim to enhance the properties of the ITZ, particularly its porosity and local hardness, by strengthening the chemical adhesion and mechanical interlocking between the rough rock surface and the cement paste. Moreover, the multi-scale coupling between micro-pore network coarsening and macro-mechanical degradation should be systematically evaluated to design resilient backfill support systems.

Additionally, considering the booming advancements in deep learning and data mining, tremendous progress has been made in recent decades in selecting constituent contents and thermal boundary parameters as key features for model inputs to predict a given set of target mechanical properties of composite structures. Through the quick extraction of valuable information, neural network models exhibit significant advantages in establishing quantitative relationships between microstructural alterations (such as pore connectivity and phase volume fractions) and the macro-physical and mechanical behaviors of the ITZ under thermal action. Future research using neural networks or physics-informed models should focus on: (1) automating ITZ segmentation and extracting microstructural features (e.g., width, porosity) from imaging data; (2) correlating phase transformations with temperature-dependent mechanical properties; and (3) predicting macroscopic fracture performance based on fundamental material and environmental inputs.

## Figures and Tables

**Figure 1:**
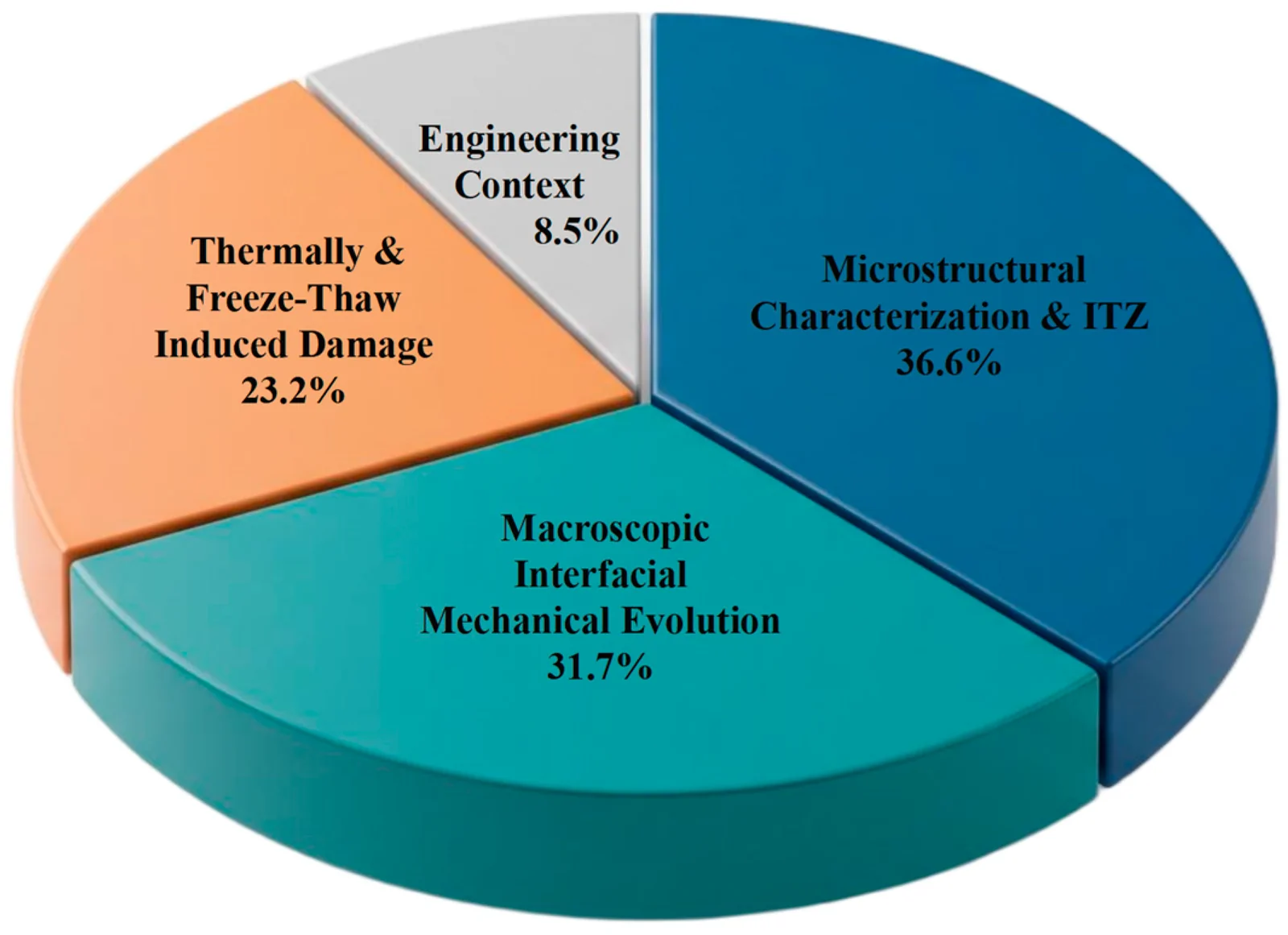
Percentage distribution of the 82 core references across four primary thematic clusters.

**Figure 2:**
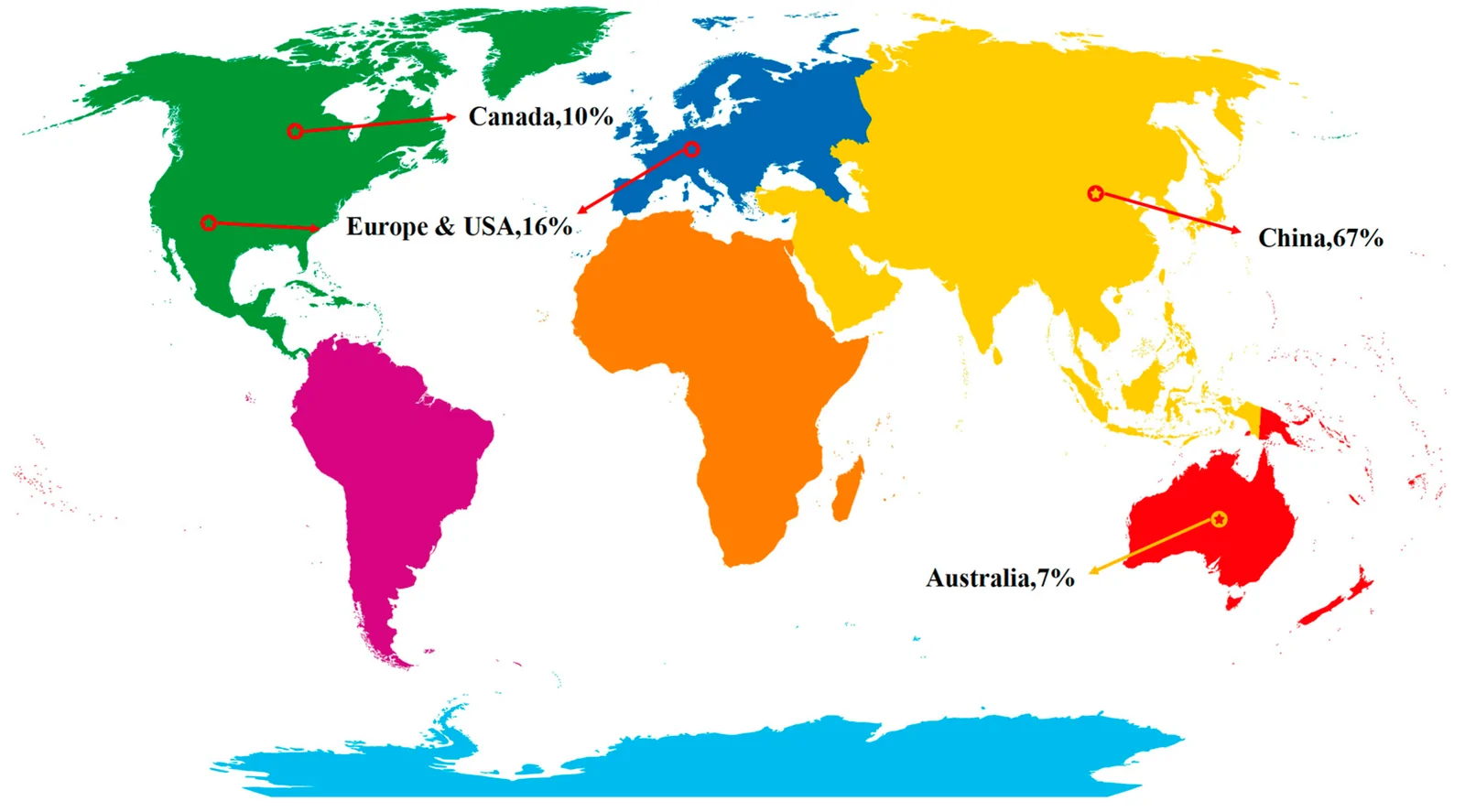
Geographic distribution of 82 references.

**Figure 3:**
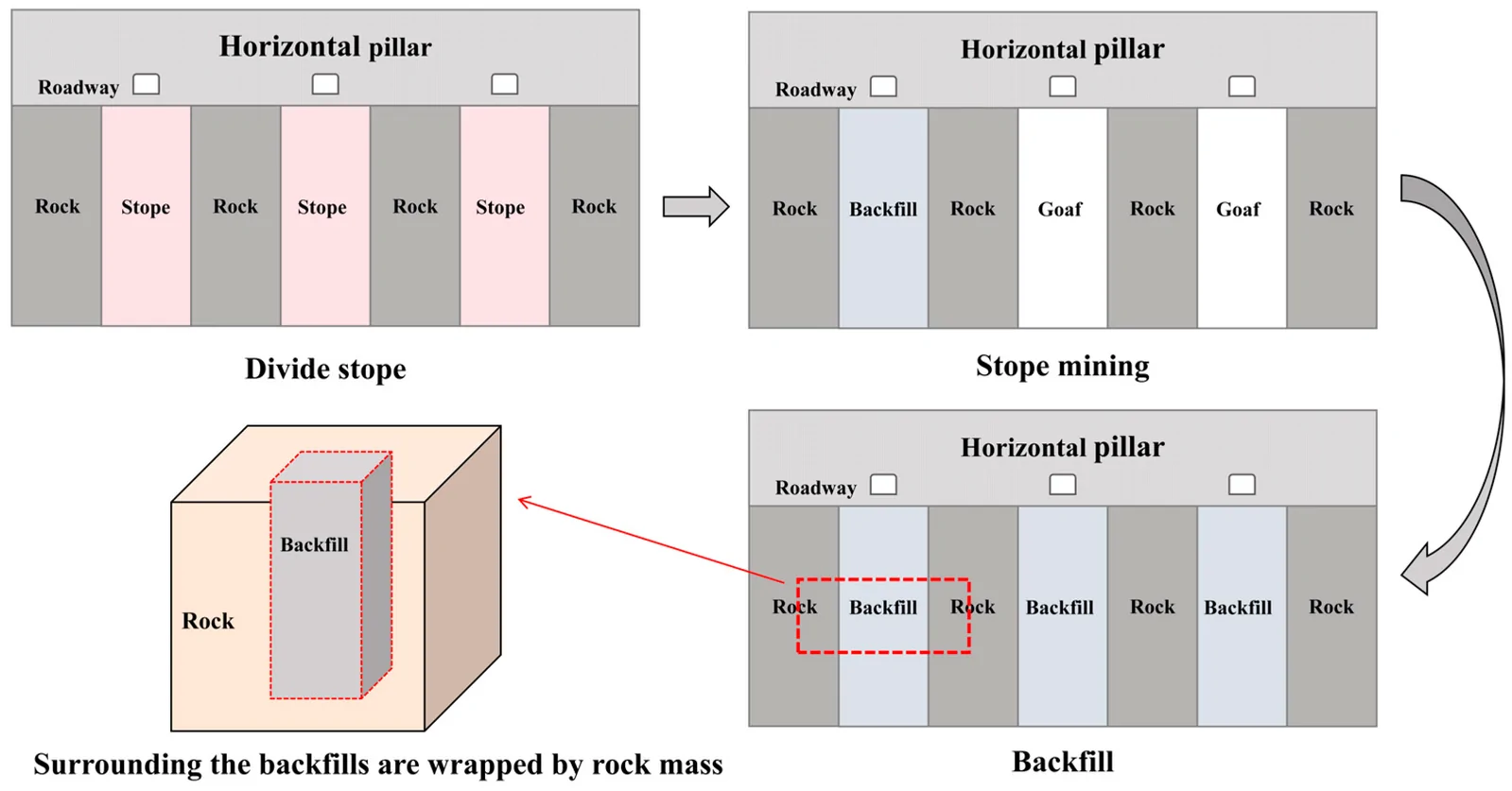
A composite structure comprising an interlaced arrangement of rock and backfill.

**Figure 4:**
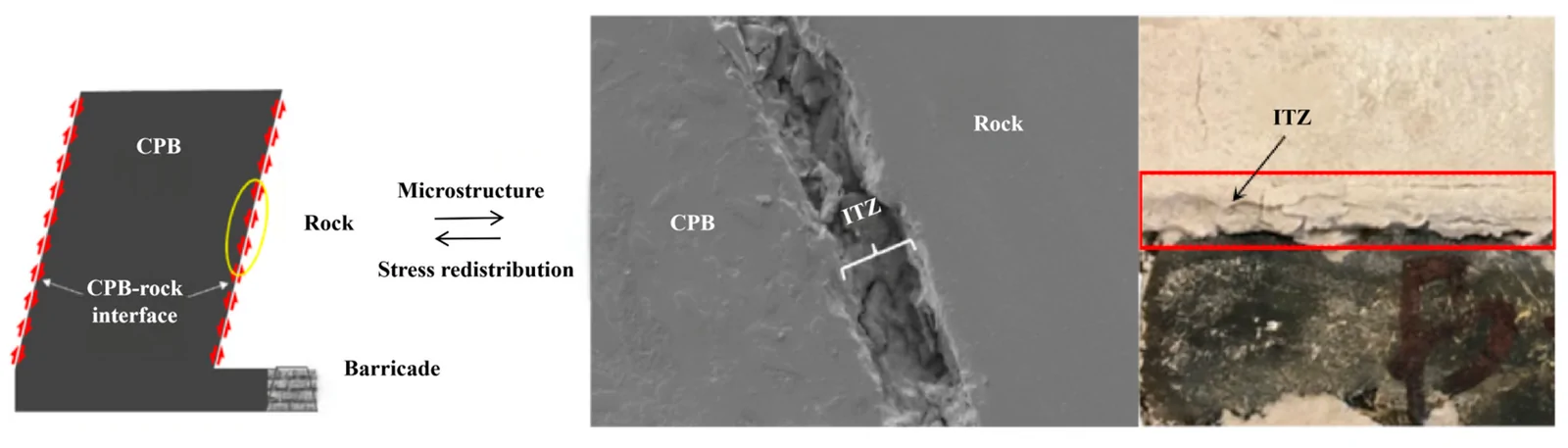
Transition zone (ITZ) between rock and backfill. Ref. [15] with permission from International Journal of Mining Science and Technology.

**Figure 5:**
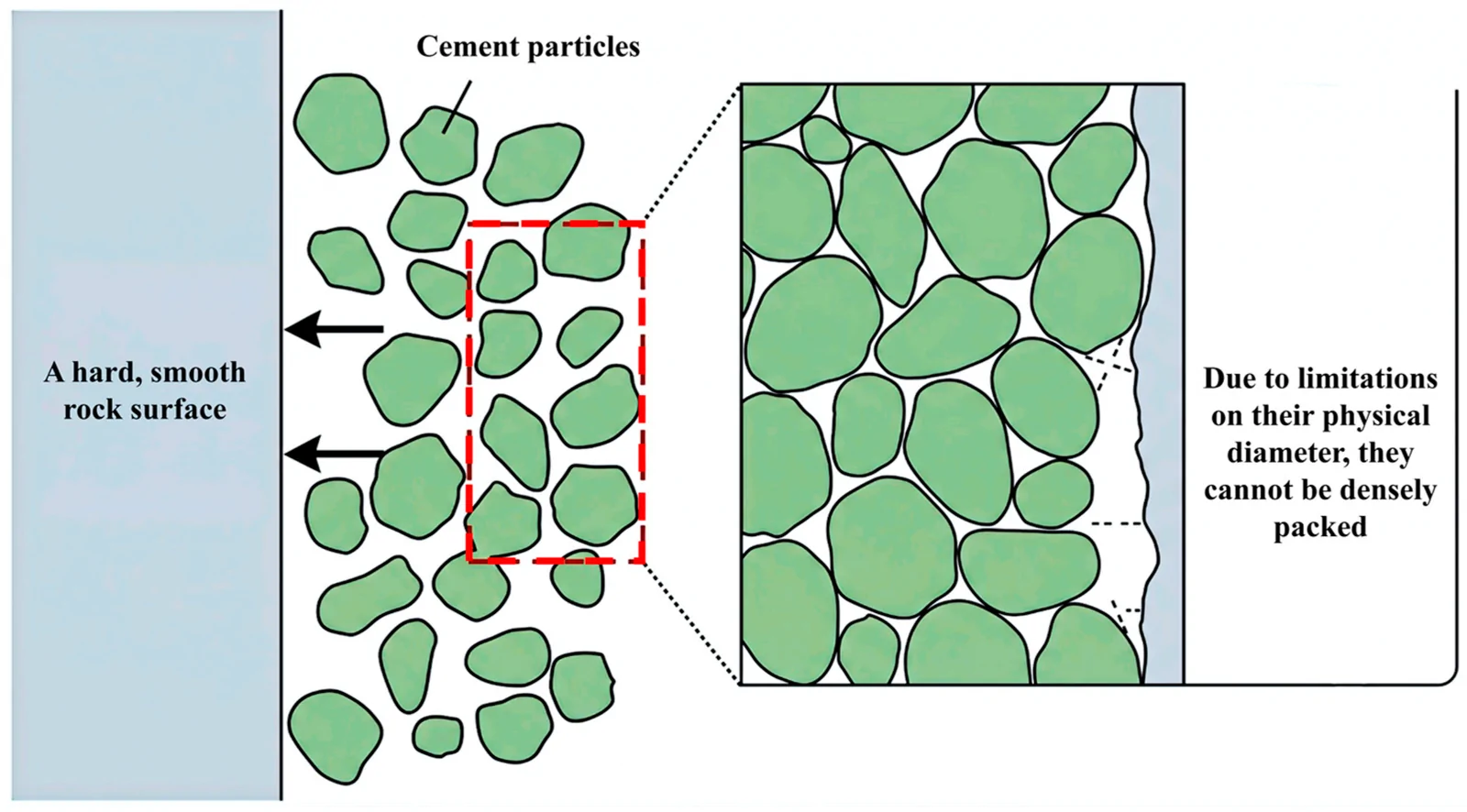
Schematic diagram of the ITZ ‘wall effect’.

**Figure 6:**
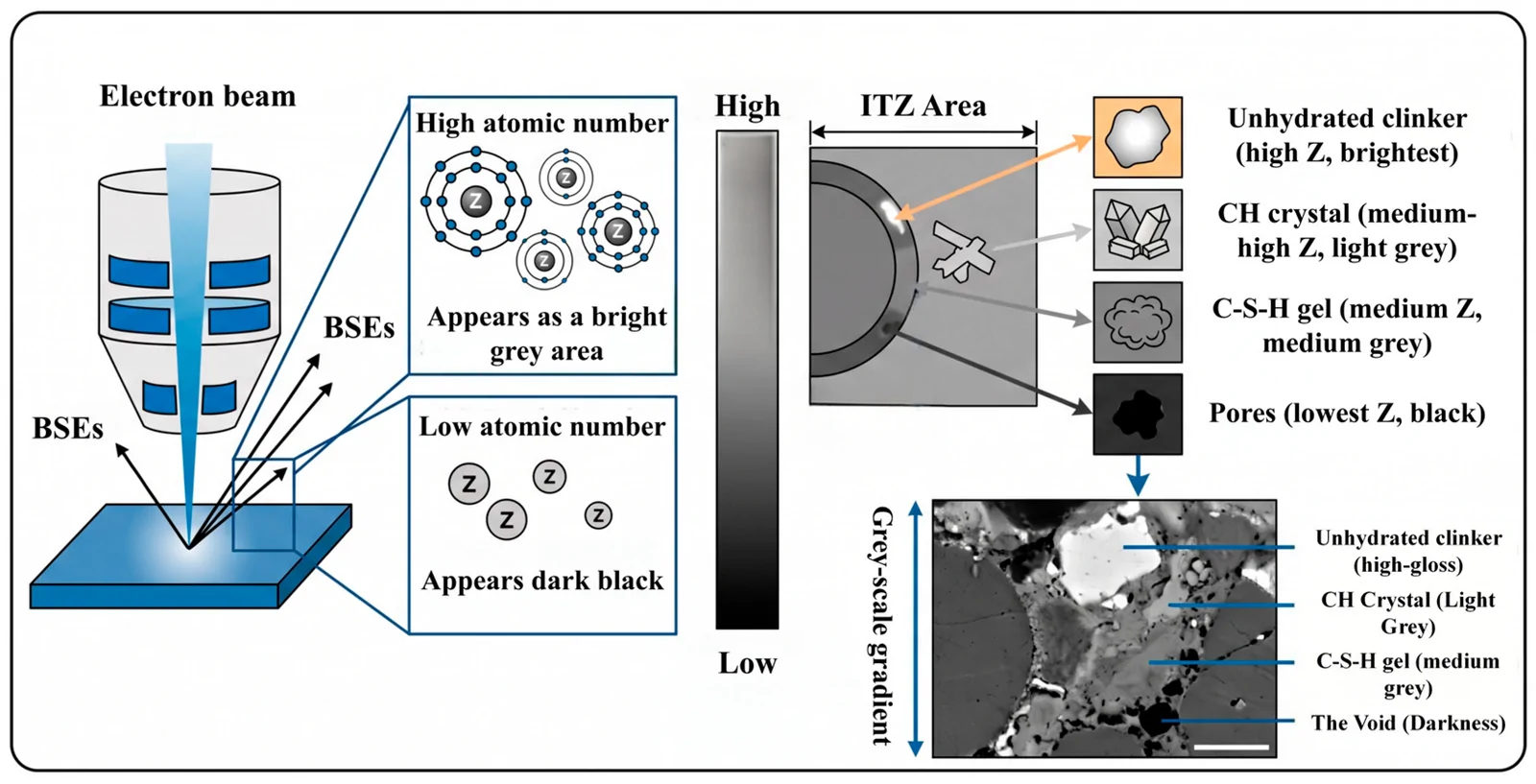
Schematic diagram of atomic number contrast imaging using BSE-SEM.

**Figure 7:**
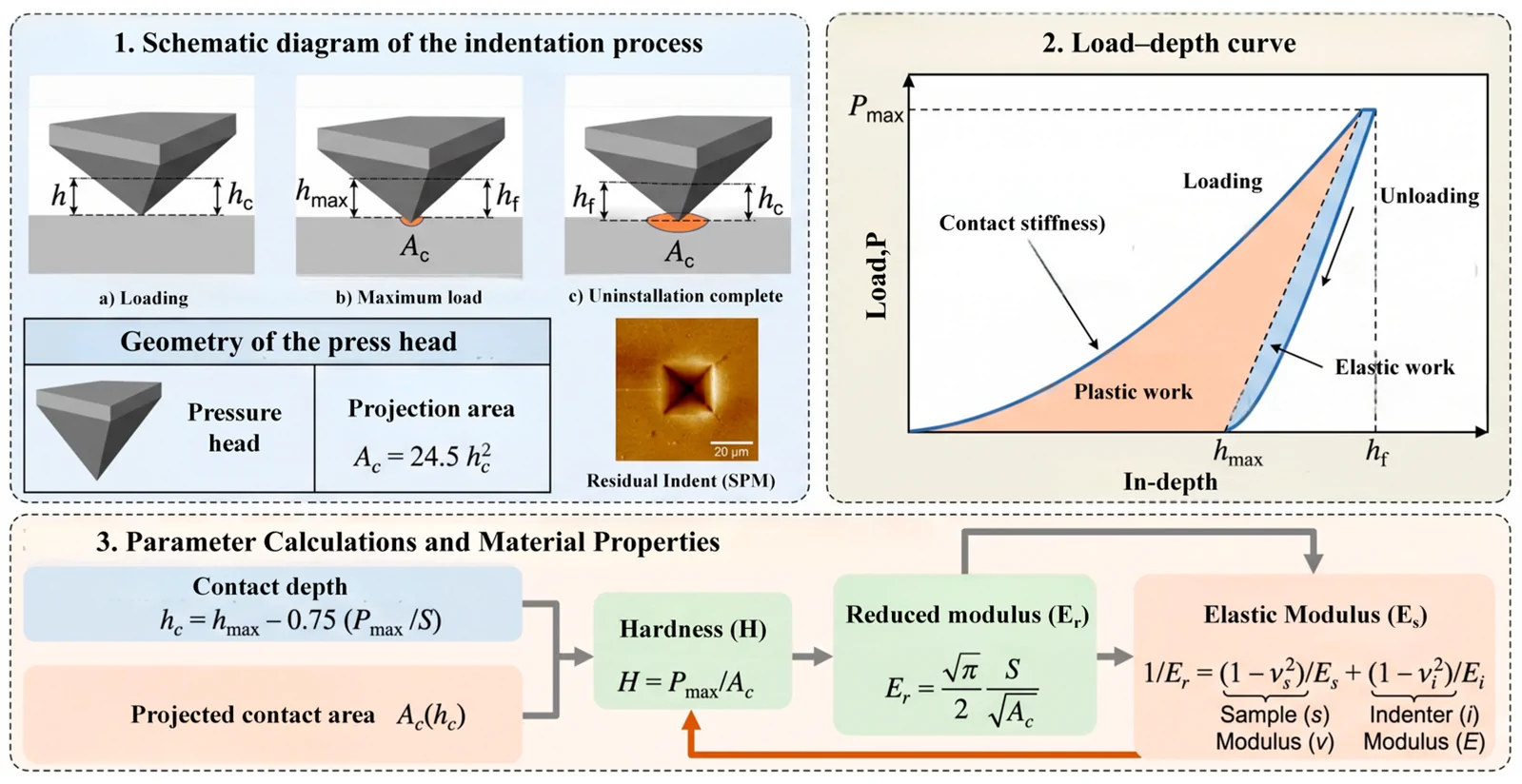
Schematic diagram of the nanoindentation technique.

**Figure 8:**
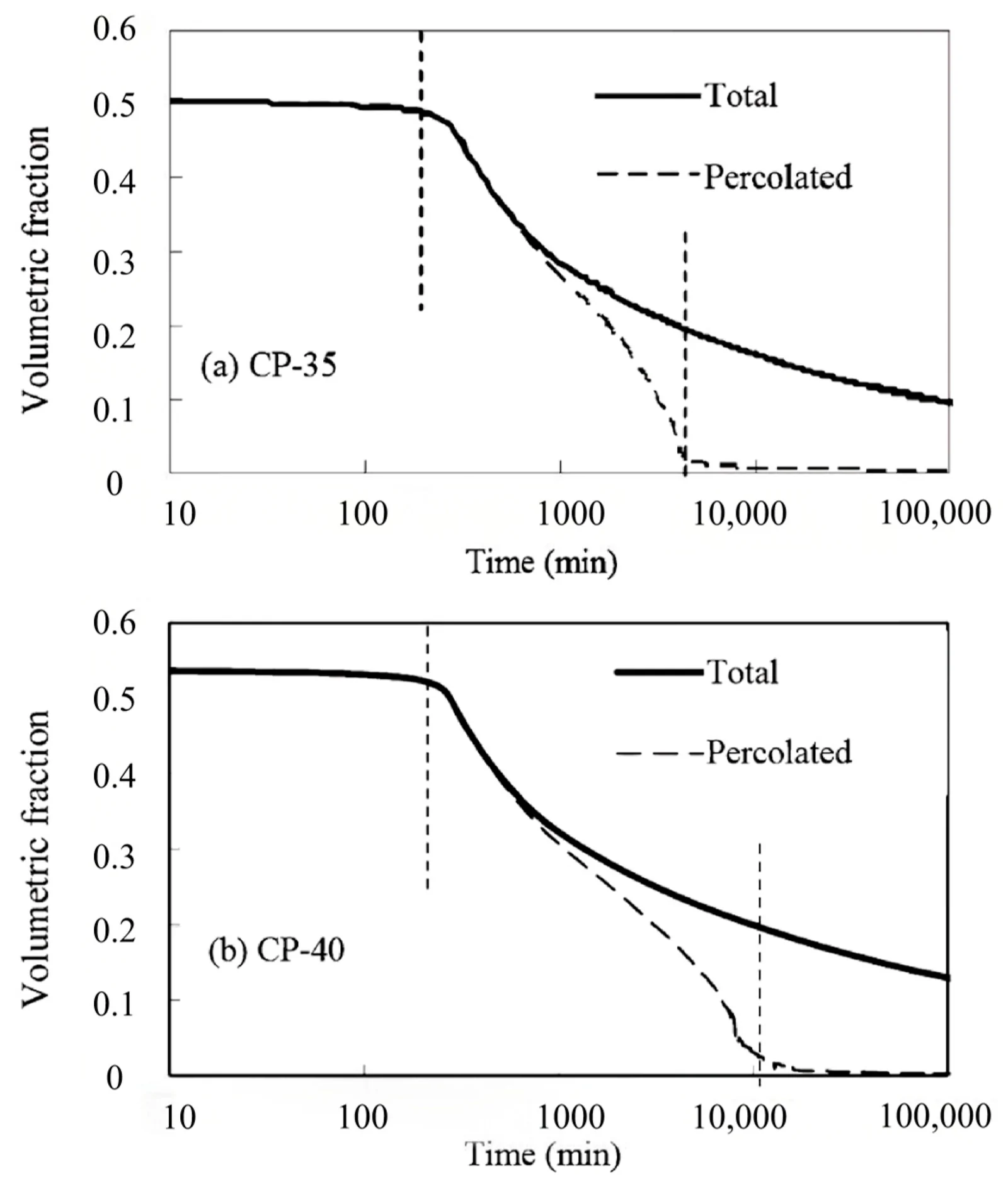
Volumetric fractions of total and percolated pores in simulated microstructure of hydrating cement paste with CEMHYD3D. Ref. [40] with permission from Cement and Concrete Research.

**Figure 9:**
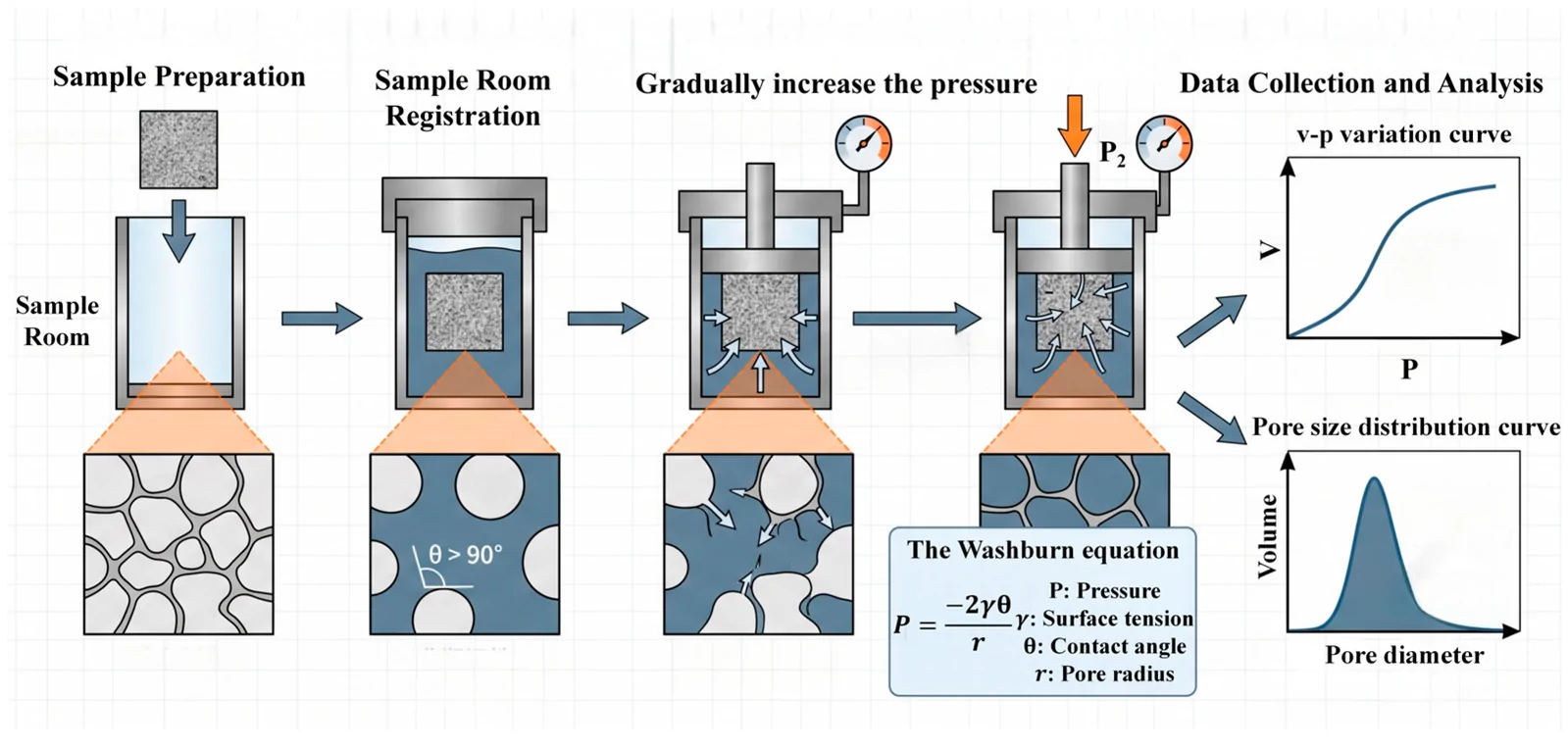
Schematic diagram of the mercury intrusion porosimetry (MIP).

**Figure 10:**
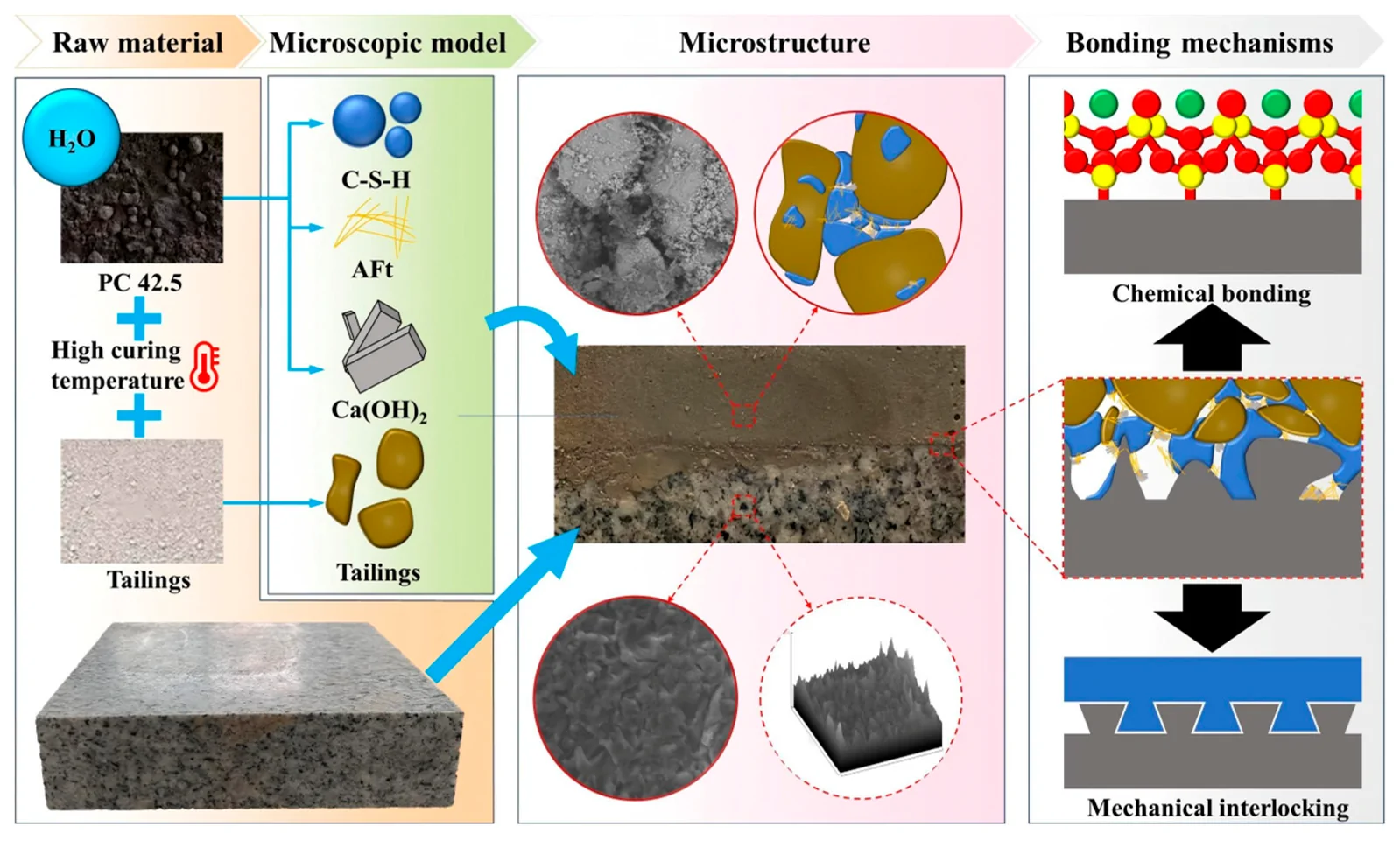
Schematic diagram of bonding mechanisms that occurred in rock–CPB interface. Ref. [51] with permission from Powder Technology.

**Figure 11:**
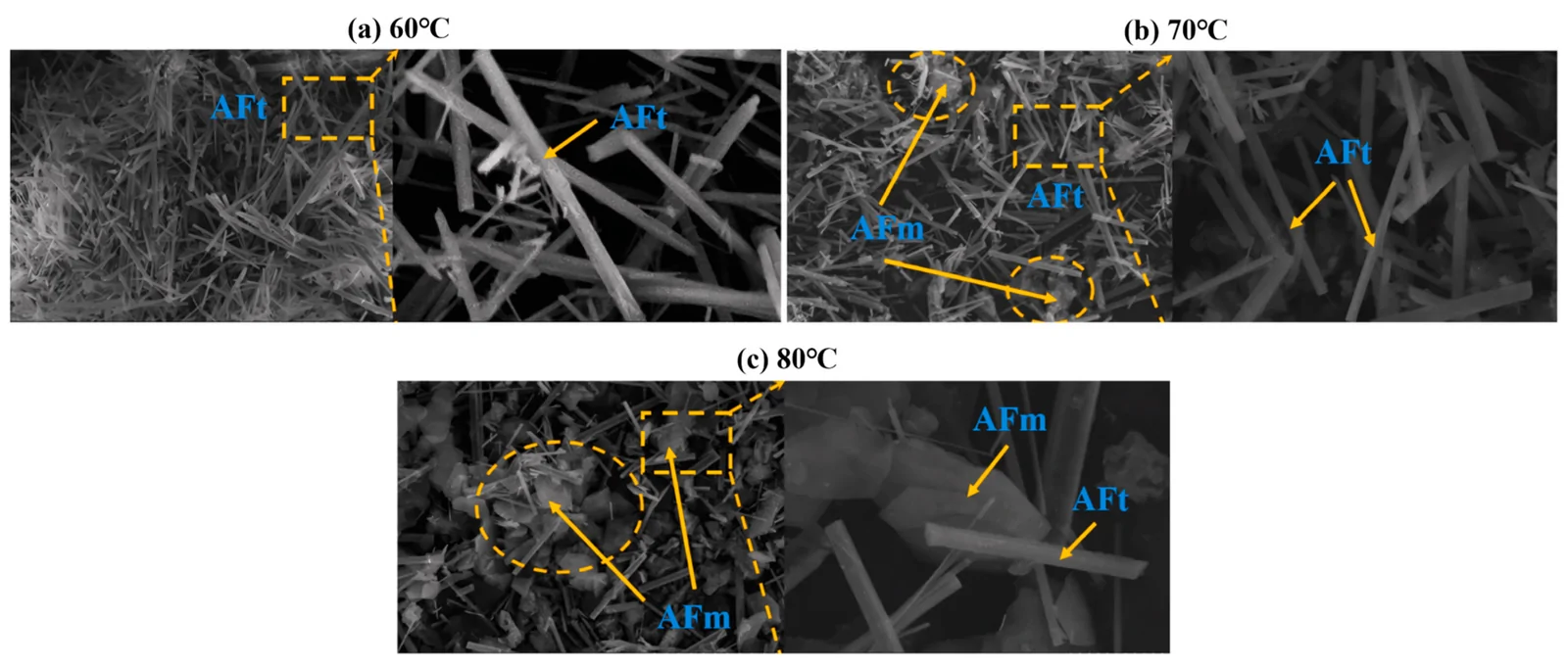
Micrographs of precipitation products formed at different liquid phase temperatures: (**a**) 60 °C; (**b**) 70 °C; (**c**) 80 °C. Ref. [53] with permission from Journal of Building Engineering.

**Figure 12:**
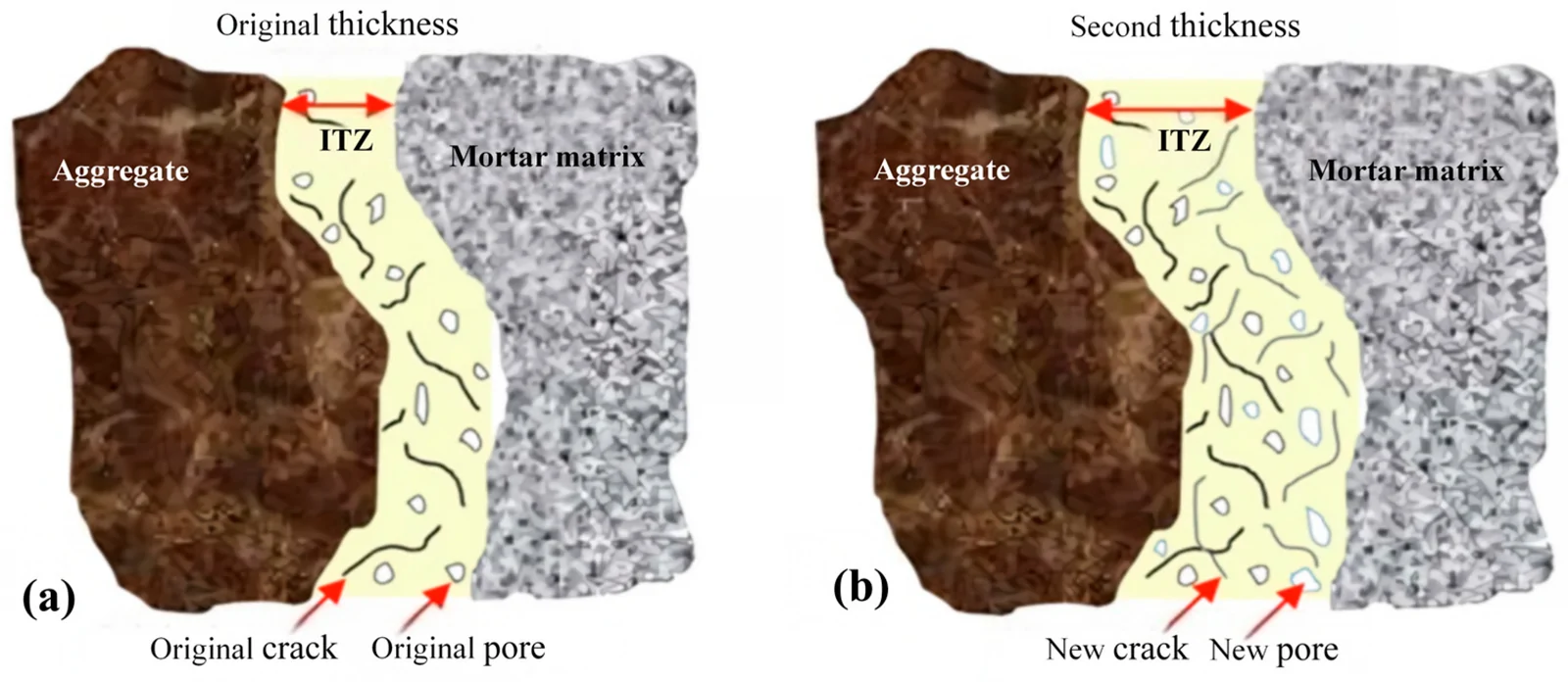
Deterioration mechanism of ITZ before and after F-T cycles. (**a**) ITZ initial state; (**b**) deterioration of ITZ after F-T cycles [61].

**Figure 13:**
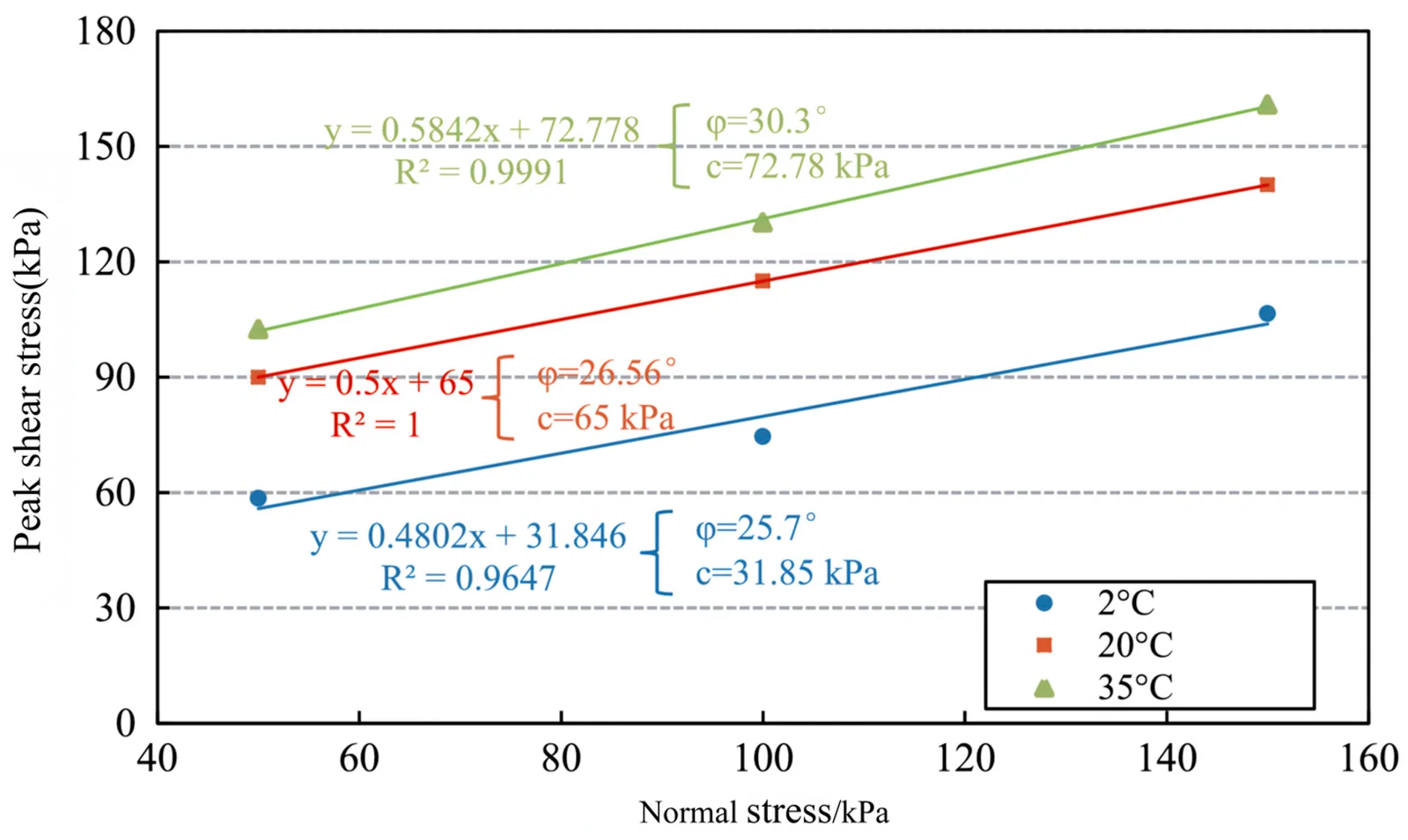
Peak shear stress vs. normal stress of CPB–rock interface (cured for 7 days). Ref. [52] with permission from International Journal of Rock Mechanics and Mining Sciences.

**Figure 14:**
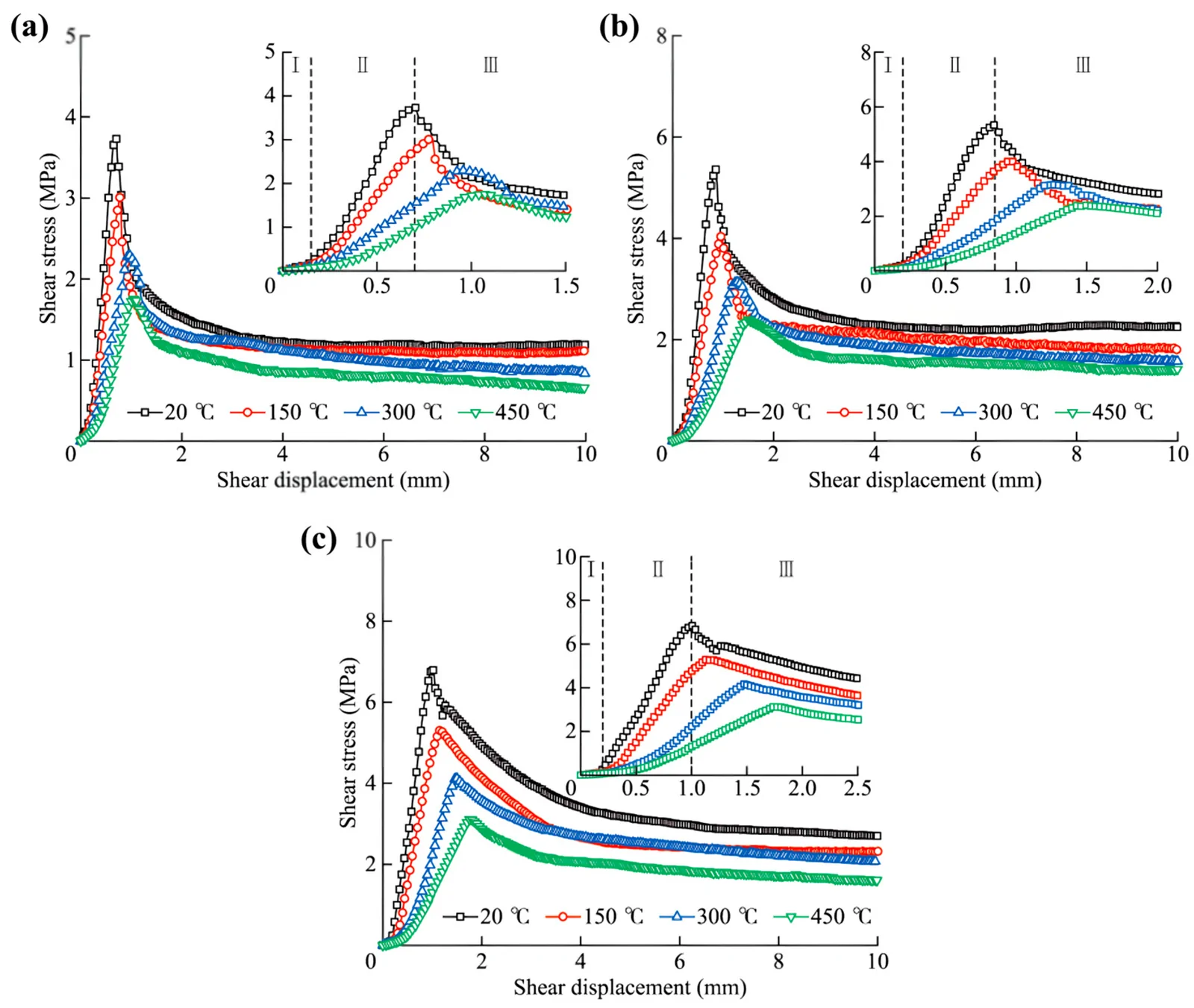
Shear stress–shear displacement at the rock–concrete interface under normal stress: (**a**) 1 MPa, (**b**) 2 MPa, and (**c**) 3 MPa. I, II, and III represent the initial compaction, linear elastic, and post-peak strain softening stages, respectively [70].

**Figure 15:**
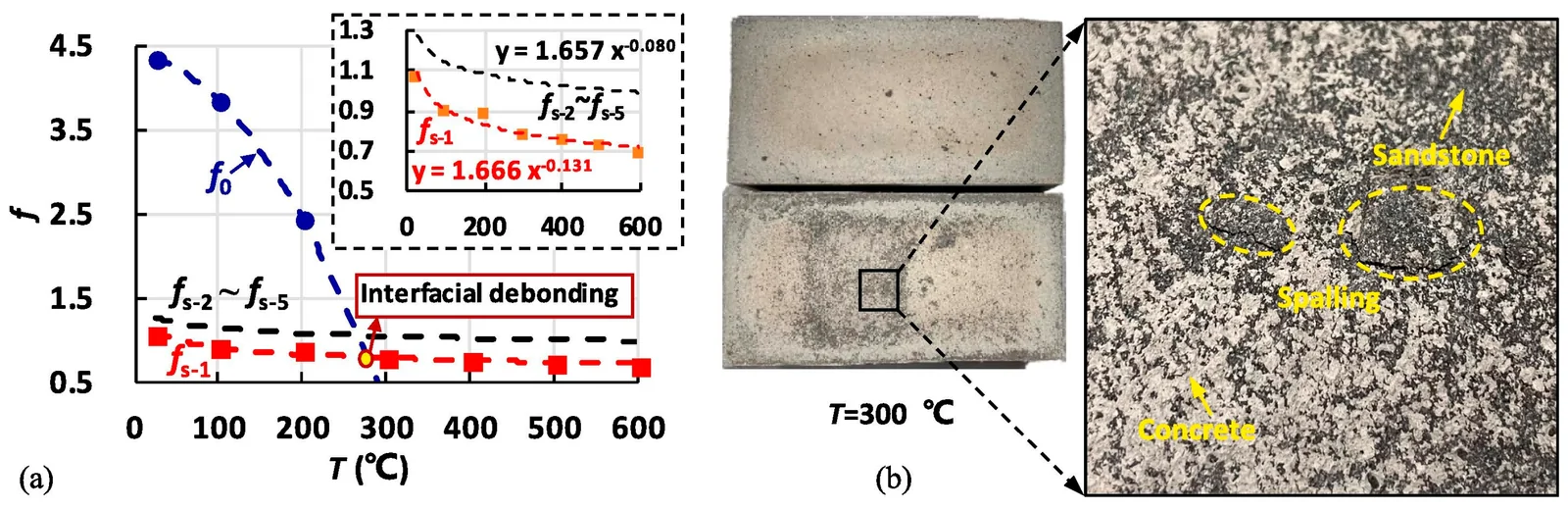
Variation in friction coefficient with increasing pre-heating temperature, (**a**) and heating-induced debonding of rock–concrete interface at T = 300 °C, (**b**). Ref. [71] with permission from Construction and Building Materials.

**Figure 16:**
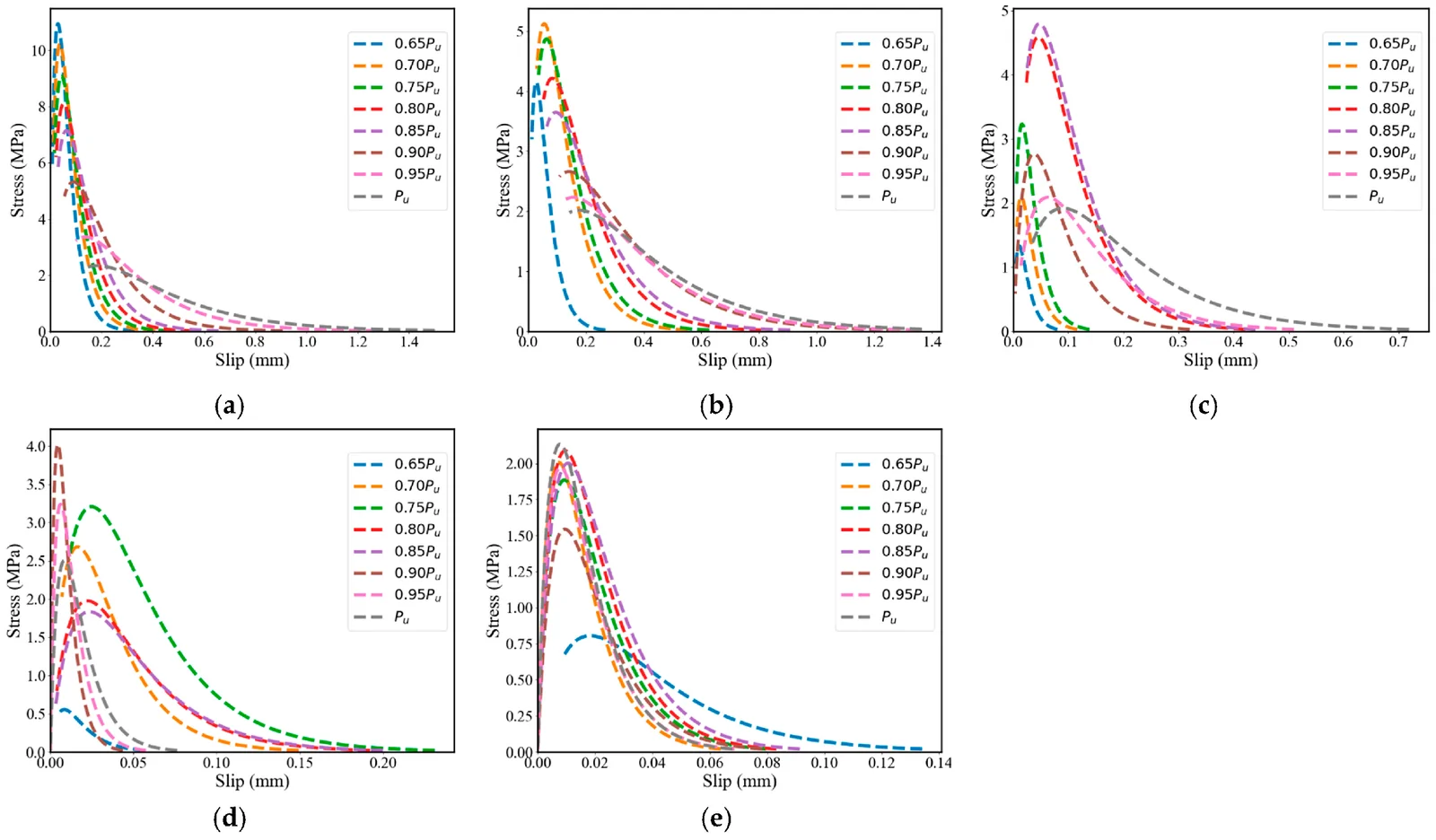
Bond-slip curves for different loads: (**a**) FT0; (**b**) FT50; (**c**) FT100; (**d**) FT150; (**e**) FT200 [76].

**Table 1:** Systematic retrieval configuration, multi-tier search queries, and eligibility criteria.

Category	Operational Specification/Criteria
Databases	Scopus, Web of Science Core Collection, ScienceDirect, SpringerLink, Taylor & Francis Online, CNKI
Search Horizon	Primary database retrieval up to 2026; backward snowballing extending to 1945 for classical theories
Tier I: Engineering Context	Geothermal gradients, deep in situ mining stresses, permafrost environments, stope support mechanics
Tier II: Characterization Methods	Analytical models, spatial resolutions, boundary artifacts for BSE-SEM, nanoindentation, X-ray CT, and MIP
Tier III: Interfacial Evolution	Boolean combination of three targeted components: Component 1 (Interfacial terms): (“interfacial transition zone” OR “ITZ” OR “paste–rock interface” OR “rock–backfill interface” OR “CPB–rock interface” OR “rock–concrete interface” OR “interfacial bond *” OR “shear behavior”) Component 2 (Matrix & Geological terms): (“cemented paste backfill” OR “CPB” OR “cemented tailings backfill” OR “backfill” OR “concrete” OR “mortar” OR “shotcrete”) AND (“rock” OR “surrounding rock” OR “host rock” OR “aggregate”) Component 3 (Thermal Boundary & Damage terms): (“temperature” OR “thermal” OR “curing temperature” OR “high temperature” OR “elevated temperature” OR “thermal damage” OR “freeze–thaw” OR “frost heave” OR “geothermal” OR “hydration heat”) Primary Search String: (Component 1) AND (Component 2) AND (Component 3).
Inclusion Criteria	(a) Original peer-reviewed research papers and authoritative review articles published in SCI/EI-indexed journals or seminal conference proceedings; (b) Studies presenting quantitative microstructural metrics (e.g., phase volume fractions, micro-porosity, pore size distributions) or interface mechanical parameters (e.g., normal bond strength, cohesive strength, internal friction angle, shear stress–displacement responses); (c) Studies providing explicit thermal boundary conditions (e.g., curing temperature protocols, target exposure temperature, heating/cooling rates, or freeze–thaw temperature amplitudes and cycle counts); (d) Seminal historical studies (identified via backward citation chaining dating to 1945) that provide the indispensable theoretical underpinnings of cement physical chemistry and frost damage; (e) Studies investigating analogous cementitious systems (e.g., rock–shotcrete, aggregate–paste, or FRP/steel–concrete interfaces) where direct rock–CPB experimental data remain sparse, provided that the underlying physical mechanisms are transferable and explicitly qualified.
Exclusion Criteria	(a) Duplicate citations across overlapping database indexes; (b) Studies investigating monolithic rock or unconfined bulk cemented backfill without an interfacial boundary; (c) Non-thermal chemical deterioration mechanisms (e.g., static sulfate attack or leaching without temperature variation); (d) Non-peer-reviewed technical reports, trade presentations, or speculative patents lacking empirical validation.

The asterisk (*) denotes a truncation wildcard used in the search strings to capture multiple word forms.

**Table 2:** Comparative analysis benchmarking the present review against major previous review papers.

Analytical Dimension	Chen et al. (2024) [11]	Wu et al. (2025) [12]	Present Review
Primary system	Structural concrete	Surrounding rock–CPB composite	Rock–CPB composite with explicit ITZ
Interfacial scale	Aggregate–paste ITZ; fiber–matrix interfaces	Planar contact; stope-scale interaction	Meso-to-micro ITZ boundary layer (15–50 μm domain) between rock and backfill
Thermal regimes	Ambient standard curing	Not systematically addressed	1. Elevated curing (2–60 °C); 2. High-temperature exposure (100–600 °C); 3. Freeze–thaw (−20 to 20 °C)
Characterization	Multi-technique comparison (SEM, nanoindentation)	Macroscopic testing overview	Critical assessment of BSE-SEM, nanoindentation, X-ray CT, MIP with resolution/artifact limits
Mechanical indicators	Interfacial fracture, bond strength	Stope-scale compression, shear	Normal bond strength, cohesion, friction angle, shear stress–displacement
Methodological novelty	-	-	Multi-tier retrieval protocol; rigorous separation of curing vs. thermal damage vs. frost action

**Table 5:** Summary of key studies on thermally induced interfacial degradation.

Ref./Year	Material System	Thermal Regime	Characterization or Test Method	Principal Findings	Limitations
Fang & Fall (2018) [52]	Direct CPB–rock	Elevated curing (2–35 °C)	Direct shear test, TGA, MIP	35 °C increases 7-day cohesion via accelerated kinetics but coarsens pores, reducing 28-day strength.	Limited to early-age curing temperatures; lacks long-term cyclic data.
Tong et al. (2016) [66]	Analogous (Shotcrete–rock)	Elevated temperature (20–100 °C)	Hot-state direct shear test	Peak shear strength is moisture-dependent, peaking at 60 °C (55% RH) and 80 °C (95% RH).	Based on shotcrete rather than CPB; limited temperature range.
Wang et al. (2023) [53]	Analogous (Cement matrix)	Elevated temperature (60–80 °C)	SEM, XRD	Identified 70–75 °C as critical window for AFt to AFm transformation.	Conducted on bulk cement matrix, not direct rock interface.
Afflerbach et al. (2023) [54]	Analogous (Ettringite-rich cement)	Thermal treatment	Mechanical testing, micro-characterization	Thermal treatment alters mechanical properties and phase composition of ettringite-rich systems.	Specific to high-ettringite formulas; lacks rock interface context.
Ma et al. (2025) [55]	Analogous (C-S-H gel)	High temperature (up to 400 °C)	In situ composition & structure investigation	Interlayer contraction near 200 °C and structural collapse by 400 °C.	Focuses on pure C-S-H phase rather than composite ITZ.
Hu et al. (2020) [67]	Analogous (Shotcrete–rock)	Heat damage & variable curing	Shear tests, constitutive modeling	Higher initial exposure temperatures amplify post-peak softening in shear behavior.	Derived from shotcrete–rock interface systems.
Chen et al. (2021) [68]	Analogous (Steel–recycled concrete)	High temperature (200–600 °C)	Residual bond testing	Residual ultimate bond strength drops by 32.8% to 79.7% from 200 to 600 °C.	Extrapolated from structural concrete with steel elements.
Zhang et al. (2021) [57,69]	Analogous (Granite–cement)	Pre-heating treatment (25–600 °C)	Direct shear, permeability tests	Peak shear strength, cohesion, and friction angles decline proportionally with temperature.	Uses granite-cement interfaces; high-temperature cooling contraction effects included.
Chen et al. (2025) [70]	Analogous (Rock–concrete)	High temperature (20–450 °C)	Residual direct shear test	Failure plane progressively migrates to the rock–concrete ITZ above 300 °C.	Based on conventional concrete rather than mine backfill.
Zhang et al. (2023) [71]	Analogous (Rock–concrete)	High temperature (up to 600 °C)	Cyclic shear testing	Dehydration forces failure transition from mixed internal and interfacial to brittle planar slip above 300 °C.	Focuses on cyclic shear of rock–concrete interfaces.
Hu et al. (2024) [72]	Analogous (Fiber shotcrete–rock)	High and variable temperature	High-temperature shear testing	Evaluated shear characteristics of fiber-reinforced shotcrete–rock interfaces under thermal action.	Uses fiber-reinforced shotcrete instead of paste backfill.
Wang et al. (2020) [73]	Analogous (Sandstone–mortar)	Freeze–thaw cycles	Direct shear test	Interfacial shear strength decreases linearly with freeze–thaw cycle counts.	Tested on mortar rather than paste backfill.
Shen et al. (2020) [74,79]	Analogous (Rock–concrete)	Freeze–thaw cycles	NMR tracking, bond model	Capillary freezing leads to rapid cohesion loss and meso-debonding at the interface.	Focuses on concrete interface; model parameters require calibration for CPB.
Kai et al. [75]/Zhang et al. (2021) [57,69]	Analogous (Concrete matrix)	Freeze–thaw cycles	Porosity and mechanical testing	Tracked porosity increases and mechanical deterioration under freeze–thaw cycles.	Evaluates bulk concrete rather than distinct ITZ.
Pan et al. (2021) [62]	Analogous (Sandstone–concrete)	Freeze–thaw cycles	Nanoindentation, direct shear	Local elastic modulus and hardness decrease, while ITZ thickness increases.	Uses concrete-sandstone specimens.
Wang et al. (2024) [77,81]	Analogous (Fiber-reinforced concrete–rock)	Freeze–thaw cycles	Shear tests	PVA fibers bridge localized microcracks to enhance shear durability under freeze–thaw.	Evaluates fiber-reinforced systems.

## Data Availability

No new data were created or analyzed in this study. Data sharing is not applicable to this article.
